# The role of m^6^A methylation in female reproductive physiology and pathology

**DOI:** 10.1016/j.gendis.2025.101755

**Published:** 2025-07-01

**Authors:** Jie Ding, Yalun He, Yangshuo Li, Shuai Sun, Wen Cheng, Jiami Huang, Chaoqin Yu

**Affiliations:** a Department of Gynaecology of Traditional Chinese Medicine, The First Affiliated Hospital of Naval Medical University, Shanghai 200433, China; b Department of Physiotherapy of Traditional Chinese Medicine, Beidaihe Rehabilitation and Recuperation Center, Joint Logistic Support Force of the People's Liberation Army, Qinhuangdao, Hebei 066000, China; c Department of Integrated Traditional Chinese and Western Medicine, Hospital of Obstetrics and Gynecology, Shanghai Medical School, Fudan University, Shanghai 200011, China; d Department of Gynaecology, Yueyang Hospital of Integrated Traditional Chinese and Western Medicine, Shanghai University of Traditional Chinese Medicine, Shanghai 200081, China

**Keywords:** Endometrial receptivity, Female infertility, Granulosa cell, Immune environment, m^6^A methylation, Oocyte

## Abstract

N6-methyladenosine (m^6^A) is a critical regulator of female reproductive physiology, yet existing reviews have focused predominantly on oocytes. The objective of this review is to systematically evaluate the regulatory effects of m^6^A throughout the pregnancy process. This review covers aspects such as oocyte maturation, granulosa cell dynamics, endometrial receptivity, immune homeostasis, and systemic adaptations, aiming to demonstrate the comprehensive regulatory capacity of m^6^A in female reproduction. Dysregulated m^6^A modifications in infertility-associated pathologies, including endometriosis, polycystic ovary syndrome, and recurrent miscarriage, are analyzed to identify mechanistic links between an epitranscriptomic imbalance and reproductive dysfunction. The key findings indicate that m^6^A is involved in the entire reproductive process and precisely coordinates stage-specific molecular programs within it, whereas aberrant methylation patterns disrupt gene networks essential for fertility. Notably, m^6^A-modifying enzymes exhibit strong potential as diagnostic biomarkers for female reproductive disorders. The synthesis of the current evidence establishes m^6^A dysregulation as a convergent pathogenic mechanism in diverse infertility etiologies, suggesting that the therapeutic modulation of m^6^A pathways could address unmet clinical needs in reproductive medicine.

## Introduction

Epigenetic modifications, which are genetic mechanisms that regulate heritable gene expression without altering the DNA sequence, involve mainly DNA methylation, histone modifications, and noncoding RNA modifications. These modifications play important roles in multiple biological processes and disease progression.^1^ Moreover, with advancements in research, chemical modifications at the RNA level, such as methylation, acetylation, and pseudouridylation, have been incorporated into the broad epigenetic regulatory system. These modifications influence RNA biogenesis, transport, functionality, and metabolism, serving as key regulatory factors in cell biology.^2^ Among these modifications, N6-methyladenosine (m^6^A), the most abundant and distinctive modification of both coding and noncoding RNAs (ncRNAs), accounts for more than 80% of RNA methylation events.^3^ m^6^A occurs across full-length transcripts but is predominantly enriched near the stop codon and within the 3′ untranslated region (3′ UTR).^4^ It participates in nearly all stages of the RNA lifecycle, including regulating transcription, maturation, translation, splicing, degradation, and stability.^5^ Advances in high-throughput sequencing technologies have significantly improved our understanding of the roles of m^6^A in regulating gene expression, cellular physiology, and disease pathogenesis.^6^, ^7^, ^8^

Due to its regulatory role in RNA synthesis, m^6^A is involved in the pathogenesis of various diseases, including obesity, diabetes, and several types of tumors.^5^^,^^9^^,^^10^ In the context of reproductive system pathology and physiological processes, m^6^A is highly important, particularly in the maturation of oocytes and sperm.^11^ According to data from the World Health Organization, approximately 1 in 6 couples of reproductive age face infertility issues worldwide. Among women of reproductive age, approximately 10%–15% suffer from infertility, making infertility a prevalent and challenging global problem.^12^ Female infertility is a complex condition involving various factors, such as abnormal ovarian function, abnormal uterine morphology or function, blocked fallopian tubes, immune system abnormalities, endocrine disorders, and unfavorable lifestyle conditions. Currently, most review articles on m^6^A modifications in gynecology focus on gynecological oncology and on oocytes, yet they lack comprehensive discussions of female infertility. Therefore, this review aims to summarize and emphasize the role of m^6^A modifications in the entire process of infertility and female reproductive system diseases. In addition, we aim to analyze the differential m^6^A modifications associated with various reproductive system diseases, with the hope of identifying common m^6^A differences that can guide subsequent diagnostic and treatment approaches.

### The mechanism and regulation of m^6^A RNA methylation

**Figure 1:**
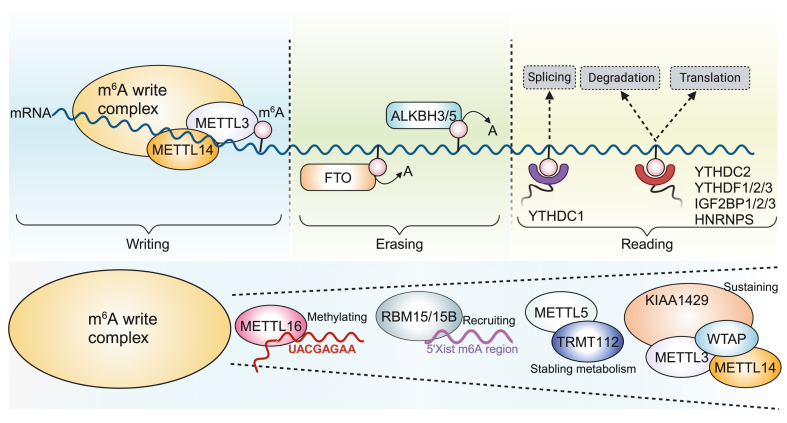
Writing, erasing, and reading in m^6^A methylation. The nuclear-localized m^6^A writer complex, consisting of METTL3 as its catalytic core along with associated regulatory subunits, mediates co-transcriptional installation of m^6^A modifications during RNA synthesis. The primary m^6^A erasers, comprising ALKBH3, ALKBH5, and FTO, are predominantly nuclear-localized enzymes that reversibly catalyze the removal of m^6^A modifications. The reader protein YTHDC1 primarily regulates RNA splicing, while other readers, including YTHDC2, YTHDF1/2/3, IGF2BP1/2/3, and HNRNPs, mediate diverse biological functions such as translational regulation, splicing modulation, and mRNA stabilization through degradation inhibition. The m^6^A writer complex comprises multiple regulatory components. METTL16 directly methylates the “UAC (m6A) GAGAA” motif and regulates SAM homeostasis. WTAP mediates METTL3-METTL14 interaction, while KIAA1429 orchestrates the balance of the METTL3/METTL14/WTAP core complex. RBM15/15B recruits the m^6^A methylation machinery to the 5′ Xist m^6^A-rich region through direct RNA binding. METTL5 forms a heterodimeric complex with the methyltransferase activator TRMT112 to maintain cellular metabolic homeostasis. METTL3/5/14/16, methyltransferase 3/5/14/16; ALKBH3/5, ALKB homologue 3/5; FTO, fat mass and obesity-associated protein; YTHDC1/2, YTH domain containing 1/2; YTHDF1/2/3, YTH domain family 1/2/3; IGF2BP1/2/3, insulin-like growth factor-2 mRNA-binding protein 1/2/3; HNRNP, heterogeneous nuclear ribonucleoprotein; SAM, S-adenosylmethionine; WTAP, Wilms tumor 1-associated protein; KIAA1429, also called VIRMA, vir-Like m^6^A methyltransferase associated, VIRILIZER; RBM15/15B, RNA binding motif protein 15/15B; TRMT112, TRNA methyltransferase activator subunit 11-2.

m^6^A methylation requires three proteins, namely, methyltransferases (writers), demethylases (erasers), and methylation recognition proteins (readers), to coregulate and perform its functions. m^6^A methylation is catalyzed by m^6^A methyltransferases, and this modification is subsequently removed by demethylases. Finally, methylation recognition proteins recognize and bind to m^6^A methylation sites to guide the translation and degradation of downstream mRNAs. “Writers” and “erasers” can dynamically catalyze RNA methylation, while the function of m^6^A is determined by “readers”. These three protein families work synergistically to participate in the process of m^6^A methylation (Fig. 1).

### Writers

“Writers” are a group of N6-adenosyl methyltransferase complexes (MTC) with methyltransferase activity that are necessary to initiate m^6^A modifications and include methyltransferase 3 (METTL3), METTL5, METTL14, METTL16, Wilms tumor 1-associated protein (WTAP), and KIAA1429 (also called VIRMA, vir-Like m^6^A methyltransferase associated, VIRILIZER). Among these complexes, METTL3 was the first m^6^A methyltransferase identified; this m^6^A methyltransferase is a key component of the catalytic process and can promote the access of adenine in RNA to methyl groups from S-adenosylmethionine.^13^ METTL5 can form a heterodimeric complex with TRNA methyltransferase activator subunit 11-2 (TRMT112) to maintain the stability of intracellular metabolism,^14^ whereas METTL16 can directly methylate the “UAC(m^6^A)GAGAA” motif and regulate S-adenosylmethionine homeostasis in an m^6^A-dependent manner.^15^ METTL14 can bind to METTL3 and form a stable heterodimer, thereby stabilizing the conformation of METTL3 to increase its catalytic activity and mediate m^6^A deposition in mammalian nuclear RNA.^16^ WTAP does not contain an obvious catalytic domain but can interact with METTL3 and METTL14, initiating their localization in nuclear speckles that are rich in pre-mRNA processing factors and enhancing the catalytic activity of m^6^A methyltransferase *in vivo*.^17^ KIAA1429, the largest known component of the m^6^A MTC, is considered a scaffold for balancing the METTL3/METTL14/WTAP core components in the RNA substrate, thereby enabling the specific m^6^A methylation of the 3′ UTR and nearby stop codons.^18^^,^^19^ In addition, other components of the m^6^A MTC, such as RNA binding motif protein 15/15B (RBM15/15B), zinc finger CCHC-type containing 4 (ZCCHC4), and zinc finger CCCH domain-containing protein 13 (ZC3H13), have been discovered. The formation of m6A in XIST and cellular mRNAs is mediated by RBM15 and RBM15B, which bind to the m6A methylation complex and recruit it to specific sites on RNA, thereby triggering methylation of adenosine nucleotides in adjacent m6A consensus motifs. Furthermore, knockdown of RBM15 and RBM15B significantly impairs XIST-mediated gene silencing.^20^^,^^21^ ZCCHC4 is responsible for the m^6^A modification of 28S rRNA and interacts with various mRNA subsets.^22^ ZC3H13 can promote m^6^A methylation and plays an important role in the nuclear localization of the ZC3H13–WTAP–VIRILIZER–HAKAI complex.^23^

### Erasers

The “erasers” category includes mainly m^6^A demethylases, such as fat mass and obesity-associated protein (FTO), ALKB homologue 5 (ALKBH5), and ALKBH3, which can reverse methylation by eliminating m^6^A modifications. FTO is located mainly in the nucleus and cytoplasm and can regulate lipid metabolism by controlling the expression of lipid-related genes.^24^ FTO, the first m^6^A demethylase discovered in eukaryotic cells, belongs to the ALKB dioxygenase family, whose process of using oxidative activity to perform the oxidative demethylation of m^6^A relies on α-ketoglutarate and Fe(II).^25^ FTO and m^6^A methylation complex components can colocalize with splicing proteins in nuclear speckles, indicating that dynamic interactions can occur between them.^10^ In addition, FTO is located near alternatively spliced exons and poly(A) sites and can preferentially bind to the pre-mRNA strand in the intronic region, thus contributing to the splicing process and playing an important role in alternative splicing.^26^ ALKBH5 is the second reported RNA demethylase in mammals and is a member of the ALKB family of dioxygenases that can mediate the repair of N-alkylated nucleobases.^27^ In addition to reversing m^6^A through oxidation, the enzymatic activity of ALKBH5 may also affect RNA metabolism, mRNA export, and the assembly of nuclear speckle processing factors. The active sites of both ALKBH5 and FTO contain the basic residues Lys132 and Arg96, which may be involved in substrate selection or the release of final products after demethylation. However, the active site of ALKBH5 is more open than that of FTO, possibly because FTO has longer NRL and C-terminal domains.^28^ In addition, another m^6^A demethylase named ALKBH3 was recently found to have a stronger effect on m^6^A in tRNA than on m^6^A in mRNA or rRNA.^29^

### Readers

“Readers” can change the fate of mRNAs by recognizing m^6^A modifications, which are responsible for the biological functions of m^6^A, such as RNA decay, splicing, and translation. The main categories include the YTH domain family, insulin-like growth factor-2 mRNA-binding protein (IGF2BP) family, heterogeneous nuclear ribonucleoprotein (HNRNP) family, eukaryotic translation initiation factor 3 (eIF3), proline-rich coiled-coil 2 A (prrc2a), and fragile X mental retardation protein (FMRP). The YTH domain family can be divided into five proteins containing homologous YTH domains, namely, YTHDC1, YTHDC2, YTHDF1, YTHDF2, and YTHDF3, whose RNA-binding domains are highly conserved and can recognize m^6^A residues in a methylation-dependent manner.^30^ IGF2BPs are a highly conserved single-stranded RNA-binding protein family that includes IGF2BP1, IGF2BP2, and IGF2BP3, and these proteins play important regulatory roles in a series of processes in the RNA life cycle, such as positioning, translation control, stability, and metabolism.^31^ The three IGF2BP proteins are very similar in domain order and spacing and comprise six typical RNA-binding domains (RBDs), namely, two RNA recognition motif (RRM) structural domains and four K homologous (KH) structural domains.^32^ HNRNPs are also a family of RNA-binding proteins that contain one or more RBDs. These binding domains can promote the broad and diverse functions of the HNRNP protein family in all stages of nucleic acid metabolism.^33^ Four unique RBDs have been identified in HNRNP proteins: the RRM, the quasi-RRM, the glycine-rich domains constituting the Arg-Gly-Gly box, and KH structural domains. These domains can be used to define protein categories through special modular structures.^33^ The HNRNP protein family members include mainly hnRNPA2B1, hnRNPC, and hnRNPG. EIF3 can directly bind to a single m^6^A modification in the 5′ UTR in the absence of cap-binding factors, thereby initiating the translation of the 43S complex.^34^ Prrc2a can stabilize the Olig2 mRNA (a transcription factor associated with oligodendrocyte specification) in an m^6^A-dependent manner by binding to the consensus GGACU motif in the Olig2 coding sequence.^35^ FMRP is encoded by the FMR1 gene (a sequence-context-dependent m^6^A reader) and binds to the m^6^A site of its mRNA target, thereby interacting with the m^6^A reader YTHDF2 in an RNA-independent manner.^36^

### The role of m^6^A in reproductive physiology

**Figure 2:**
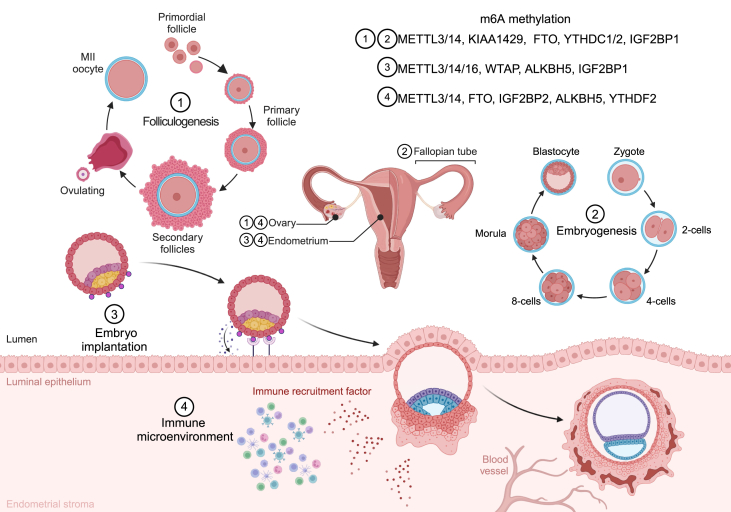
Functional roles of m^6^A modification in reproductive physiology. Successful pregnancy requires coordinated physiological processes, with m^6^A participating in critical stages: i) Folliculogenesis: m^6^A dynamically regulates oocyte-granulosa cell crosstalk during follicular maturation from primordial follicles to metaphase II (MII) oocytes, mediated by key regulators including METTL3, METTL14, KIAA1429, FTO, YTHDC1, YTHDC2, and IGF2BP1. ii) Embryogenesis: Following fertilization, m^6^A governs epigenetic modulation of zygotic development from the 2-cell stage through blastocyst formation. iii) Embryo implantation: Endometrial receptivity, a pivotal determinant of implantation success, is enhanced by m^6^A-mediated homeostatic control of hormonal metabolic signaling, involving METTL3, METTL14, METTL16, WTAP, and IGF2BP2. iv) Immune microenvironment: Embryo-derived immunomodulatory factors recruit immune cells to coordinate developmental processes. m^6^A regulators such as METTL3, METTL14, IGF2BP2, ALKBH5, and YTHDF2 modulate T cell/NK cell activities during immune adaptation. METTL3/14/16, methyltransferase 3/14/16; ALKBH5, ALKB homologue 5; FTO, fat mass and obesity-associated protein; YTHDC1/2, YTH domain containing 1/2; WTAP, Wilms tumor 1-associated protein; KIAA1429, also called VIRMA, vir-Like m^6^A methyltransferase associated, VIRILIZER; IGF2BP1/2, insulin-like growth factor-2 mRNA-binding protein 1/2; YTHDF2, YTH domain family 2; NK, natural killer.

Infertility is defined as the inability to conceive after 12 months of unprotected sex with the same partner. Approximately 85% of infertile couples have a clear cause of infertility. When male factors are excluded, female infertility can be attributed to ovulatory dysfunction, diminished ovarian reserve, tubal factors, and uterine/cervical factors. Pregnancy is a complex physiological process involving a series of events, including fertilization, embryo implantation, embryo development, hormone maintenance, and fetal development, which involve multiple factors, namely, oocyte development, granulosa cells (GCs), endometrial receptivity, the immune system, and physical conditions. The m^6^A modification influences these aspects and thus has a substantial effect on fertility (Fig. 2).

### m^6^A and oocyte development and maturation

Oocyte development is a complex and highly regulated cellular differentiation process that encompasses two main stages: follicle development and oocyte maturation.^37^ Primitive oocytes divide and develop into primary follicles with the support of surrounding cells. Primary follicles undergo rapid growth and differentiation, giving rise to the activated precursor of a mature follicle, which further envelops the oocyte.^38^ During oocyte development, the cellular volume of the oocyte increases, and changes in organelle development occur. Moreover, intense metabolic activity within the oocyte cytoplasm simultaneously promotes coordinated evolution of the nucleus, mitochondria, and cytoplasm, providing the foundation for fertilization and embryo development.^39^ Recent studies have revealed the critical role of the m^6^A modification in oocyte development. The overall summary of this step is shown in Table 1.

**Table 1:** Roles of m6A protein machineries and biological mechanisms exerted in oocyte.

Regulators	Role	Mechanism	Authors [Ref.]
**METTL3**	The quality of oocytes with conditional knockout of Mettl3 and Gdf9 is reduced.	m6A modification regulates the translation and stability of RNA during the transition from MII stage oocytes to embryos, affecting the functionality of oocytes.	Yao et al., 2023 ^30^
	Deletion of Mettl3 leads to defects in follicle development and abnormal ovulation.	Depleting Mettl3 results in DNA damage in oocytes. Mettl3 can target Itsn2 to enhance its stability, thereby affecting oocyte meiosis.	Mu et al., 2021 ^31^
	Interfering with Mettl3 RNA in oocytes results in defects in the meiotic spindle during oocyte maturation and impediments in the extrusion of the first polar body.	The dependence on CCR4 exerts a negative regulatory role in the process of gene decay adenylation in systems lacking TATA boxes, further leading to mRNA accumulation in oocytes.	Zhu et al., 2022 ^32^
	Mettl3 ensures the normal development of the embryo after fertilization in oocytes.	During the activation of the zygotic genome, METTL3 deposits m6A on mRNA and ensures the degradation of these mRNAs after the two-cell stage, including Zscan4 and MERVL.	Wu et al., 2022 ^33^
	Knocking out Mettl3 in female germ cells severely inhibits oocyte maturation and leads to defects in the transition from maternal to zygotic stages.	Knocking out Mettl3 reduces mRNA translation efficiency.	Sui et al., 2020 ^34^
**METTL14**	Reducing METTL14 can enhance the ability of porcine oocytes to undergo meiotic division, maturation, and development.	N/A	Yu et al., 2018 ^35^
**KIAA1429**	The absence of KIAA1429 leads to defects in follicle development.	The absence of KIAA1429 results in abnormal RNA metabolism in the oocyte nucleus, leading to the inability of fully-developed oocytes to undergo nuclear envelope breakdown and, consequently, the loss of their ability to initiate meiosis.	Hu et al., 2020 ^36^
**FTO**	Depletion of FTO leads to defects in oocyte development.	Depletion of FTO leads to a significant decrease in LINE1 RNA expression in GV and MII oocytes, and FTO−/− MII oocytes exhibit noticeable chromosome misalignment and increased spindle collapse.	Wei et al., 2022 ^37^
**YTHDC1**	Deficiency in YTHDC1 results in widespread selective splicing defects in oocytes.	Deletion of YTHDC2 reduces the proportion of STRA8-positive oocytes and the ratio of oocytes in the zygotene and pachytene stages.	Kasowitz et al., 2022 ^38^
**YTHDC2**	Knockout of YTHDC2 results in reproductive disorders in mice.	N/A	Zeng et al., 2020 ^39^

Yao et al conducted single-cell m^6^A sequencing to analyze the m^6^A methylome and transcriptome of individual oocytes and blastomeres during the cleavage stage of embryonic development. These findings reveal that m^6^A deficiency leads to abnormal RNA clearance, resulting in decreased oocyte quality. Furthermore, they show that m^6^A regulates RNA translation and stability during the transition from metaphase II-stage oocytes to embryos, thereby impacting oocyte functionality. Additionally, m^6^A-dependent asymmetry was observed in the transcriptomes of two-cell embryos, indicating the crucial regulatory role of m^6^A in oocyte and embryonic development.^40^ Further investigation of Mettl3 conditional knockout in oocytes reveals that Mettl3 can target intersectin 2 (Itsn2), increasing its stability and thereby impacting oocyte meiosis. The depletion of Mettl3 leads to DNA damage in oocytes, resulting in defective follicle development and impaired ovulation.^41^ Another study reveals that interference with the Mettl3 RNA in oocytes causes defects in the spindle apparatus during oocyte maturation, as well as obstacles in the extrusion of the first polar body. The underlying mechanism involves the CCR4-dependent negative regulation of gene decay adenylation in systems lacking a TATA box, leading to mRNA accumulation in oocytes and hindering their maturation process.^42^ In addition, METTL3 deposits m^6^A on mRNAs, including zinc finger and SCAN domain containing 4 (Zscan4) and murine endogenous retrovirus-L (MERVL), during zygotic genome activation and facilitates the subsequent degradation of these mRNAs after the two-cell stage to ensure proper embryonic development after fertilization.^43^ Furthermore, studies have shown that knocking out Mettl3 in female germ cells decreases the mRNA translation efficiency, inhibiting oocyte maturation and resulting in defects during the transition from the maternal stage to the zygotic stage.^44^ In addition to METTL3, animal studies have shown that ascorbic acid reduces the levels of Mettl14 in porcine oocytes, increasing their ability to undergo meiotic maturation and development.^45^ However, the specific downstream mechanisms regulated by Mettl14 have not been explored. The absence of KIAA1429 results in abnormal RNA metabolism in perinucleolar oocytes, and fully developed perinucleolar cells fail to undergo perinucleolar breakage, leading to a loss of the ability to recover from meiotic division and defects in follicle development.^46^

In terms of demethylases, Wei and colleagues show that FTO-mediated demethylation maintains the abundance of the long interspersed nuclear element-1 (LINE1) RNA in mouse embryonic stem cells. This process helps promote local chromatin accessibility and activates genes containing LINE1 elements. Further investigations reveal that the depletion of FTO leads to a significant decrease in LINE1 RNA expression in germinal vesicle- and metaphase II-stage oocytes. FTO^−/−^ metaphase II-stage oocytes exhibit notable chromosomal misalignment and increased spindle collapse, resulting in defects in oocyte development.^47^

YTHDC1, an m^6^A reader, plays a crucial role in pre-mRNA transcriptional processing in oocyte nuclei. Oocytes lacking YTHDC1 experience arrested development at the primordial follicle stage, leading to widespread selective adenosine methylation and altering the length of the 3′ UTR. Additionally, YTHDC1 deficiency leads to extensive selective splicing defects in oocytes, possibly involving the pre-mRNA 3′ end processing factors, cleavage and polyadenylation specific factor 6 (CPSF6), serine/arginine rich splicing factor 3 (SRSF3), and SRSF7.^48^ However, further research is needed to fully understand these connections. On the other hand, knockout of the YTHDC2 gene reduces the proportion of oocytes stimulated by retinoic acid 8 (STRA8)-positive oocytes and the proportion of oocytes in the zygotene and pachytene stages, resulting in reproductive abnormalities.^49^

The m^6^A modification is intimately involved in the development of oocytes and is associated with various abnormalities, such as chromosomal abnormalities in oocytes, abnormal RNA metabolism, meiotic division defects, splicing defects, and an impaired transition of the oocyte to the embryo. However, the mechanism by which the m^6^A modification affects the development of oocytes at each stage is still unclear and is limited by the difficulty of directly studying early-stage oocytes, especially in humans.

In summary, m^6^A methylation plays a multifaceted role in oocyte development and is pathologically associated with chromosomal abnormalities, RNA metabolic dysregulation, meiotic defects, splicing aberrations, and a compromised embryonic transition. However, critical knowledge gaps persist in the current research. First, the precise mechanism of the m^6^A modification during early oogenesis, particularly in primordial follicle activation, remains elusive, as most investigations have focused on the maturation phase. Second, the downstream pathways of specific m^6^A regulators require clarification; for example, the precise downstream targets responsible for the enhanced meiotic competence and developmental potential observed in METTL14-deficient oocytes have not been identified, with existing studies limited to phenotypic observations. Third, as a dynamic, reversible modification, an m^6^A imbalance likely involves multifactor dysregulation. The spatiotemporal coordination between methyltransferases and demethylases in maintaining oocyte development necessitates a systematic exploration, as current research has predominantly examined individual components. Finally, translational challenges persist between animal models and human applications. The unique regulatory characteristics of m^6^A in human oogenesis and their clinical implications demand comprehensive validation, with ethical constraints potentially impeding related investigations.

### m^6^A and GC function

GCs are closely associated with oocytes, as they envelop and provide nourishment for their development, leading to the formation of follicles. During follicle maturation, GCs provide growth factors and nutrients to promote the development and maturation of oocytes. After ovulation, GCs differentiate into luteal cells, which continue to support and nourish the embryo.^50^ The close collaboration between GCs and oocytes is crucial for their development and maturation, ensuring normal fertilization and implantation. The m^6^A modification also serves as a regulatory mechanism controlling the proliferation and functionality of GCs. Cao et al report that mRNA m^6^A modification is abundant in pig GCs during development from small to large follicles and is enriched primarily around the protein-coding regions of transcripts. The enrichment analysis suggests that m^6^A modification-related genes are strongly involved in regulating steroidogenesis and folliculogenesis. Sequencing of human ageing ovarian GCs reveals that genes, such as budding uninhibited by benzimidazoles-1B (BUB1B), polyhomeotic homolog 2 (PHC2), topoisomerase IIα (TOP2A), discoidin domain receptor 2 (DDR2), Krüppel-like factor 13 (KLF13), and ryanodine receptor 2 (RYR2), contain the m^6^A modification, contributing to declining ovarian function.^51^ The overall summary of this part is shown in Table 2.

**Table 2:** Roles of m6A protein machineries and biological mechanisms exerted in granulosa cell.

Regulator	Role	Mechanism	Authors [Ref.]
**FTO**	The lack of FTO leads to abnormal senescence of GCs, resulting in ovarian aging.	Under oxidative stress conditions, decreased FTO in GCs leads to abnormal upregulation of FOS expression.	Sun et al., 2021 ^42^; Ding et al., 2021 ^43^; Jiang et al., 2021 ^44^
	FTO can influence the proliferation, apoptosis, and insulin resistance of GCs.	FTO can also increase the stability of FLOT2 mRNA to promote FLOT2 expression. FTO can co-regulate MNT expression with YTHDF2 and alleviate GCs apoptosis and oxidative stress by activating the AKT/Nrf2 pathway.	Ding et al., 2022 ^45^
**IGF2BP1**	Downregulation of IGF2BP1 affects the vitality, proliferation, cell cycle, and cellular senescence of GCs.	IGF2BP1 can enhance the stability of MDM2 mRNA.	Mu et al., 2022 ^46^
**METTL3**	Depletion of METTL3 disrupts the cell cycle of GCs, resulting in increased apoptosis and inhibited cell proliferation and hormone secretion.	METTL3 may regulate the cell cycle of GCs by mediating the degradation of AURKB mRNA through the YTHDF2 pathway.	Liu et al., 2023 ^47^
	The decrease in METTL3 levels leads to an upregulation of autophagy in GCs.	The decrease in METTL3 levels can significantly increase the mRNA levels of autophagy key gene ULK1, thereby leading to an upregulation of autophagy in GCs and causing follicular atresia.	Li et al., 2023 ^48^
	Butyric acid can inhibit the expression of METTL3 and improve the inflammatory state of GCs.	Inhibiting the expression of METTL3 results in a decrease in FOSL3 m6A methylation level and mRNA expression, which can subsequently downregulate the expression of NLRP3 protein, IL-6, and TNF-α in GCs.	Liu et al., 2023 ^49^

Several studies have revealed that the level of FTO in GCs decreases significantly during ovarian ageing, as observed in both clinical and animal samples.^52^^,^^53^ Jiang et al further show that decreased FTO levels in GCs under oxidative stress lead to an abnormal increase in FOS expression, contributing to the abnormal ageing of GCs and subsequent ovarian ageing.^54^ Moreover, FTO can increase the stability of the flotillin 2 (FLOT2) mRNA, promoting FLOT2 expression and thereby influencing GC proliferation, apoptosis, and insulin resistance. Additionally, FTO can coregulate the expression of MAX network transcriptional repressor (MNT) with YTHDF2, alleviating GC apoptosis and oxidative stress by activating the protein kinase B (AKT)/nuclear factor erythroid 2-related factor 2 (Nrf2) pathway.^55^ In cases of ovarian dysfunction caused by oxidative stress, the m^6^A reader protein IGF2BP1 is significantly down-regulated in GCs. IGF2BP1 can regulate GC viability, proliferation, the cell cycle, and cellular ageing by increasing the stability of the mouse double minute 2 (MDM2) mRNA.^56^

Animal studies have revealed that during oocyte maturation, METTL3 expression increases in GCs, and the cell cycle is disrupted when METTL3 is deleted in these cells, leading to increased apoptosis, the inhibition of cell proliferation, and hormone secretion. Mechanistically, METTL3 may regulate the cell cycle in GCs by mediating the degradation of the Aurora kinase B (AURKB) mRNA through the YTHDF2 pathway.^57^ Additionally, the m^6^A modification in GCs plays a role in follicular atresia. The level of METTL3 in progressive atretic follicle GCs was significantly lower than that in healthy follicle GCs, whereas FTO levels were significantly higher. Research has shown that a decreased level of METTL3 can significantly increase the mRNA level of the autophagy-related gene unc-51-like kinase 1 (ULK1), leading to increased autophagy in GCs and subsequent follicular atresia.^58^ Liu et al report that butyric acid can inhibit the expression of the methyltransferase METTL3, resulting in decreased m^6^A methylation and expression of the FOS-like 3 (FOSL3) mRNA, thereby down-regulating the levels of the NLR family pyrin domain containing 3 (NLRP3) protein and inflammatory cytokines (interleukin-6/IL-6 and tumor necrosis factor-alpha/TNF-α) in GCs and improving their inflammatory state.^59^

In summary, the m^6^A modification affects GC function by regulating pathways such as those involved in steroid synthesis and autophagy. Under oxidative stress or inflammatory conditions, it mediates abnormal cell proliferation, apoptosis, and inflammatory factor expression, ultimately impairing GC and oocyte functions. However, current research has yet to clarify how m^6^A directly influences gap junctions and material exchange between GCs and oocytes. In existing studies, GCs are often isolated from oocytes for a separate analysis, compromising the integrity of the research. Additionally, while current models rely heavily on cell lines such as KGN cells, *in vivo* animal experiments and large-scale clinical sample studies are noticeably lacking, necessitating further validation and expansion of the experimental approaches.

### m^6^A and endometrial receptivity

The endometrium is indispensable for successful reproductive processes because it creates a favorable environment for the implantation of a fertilized embryo, supplies essential oxygen and nutrients to facilitate embryonic development, and produces various hormones critical for maintaining a healthy pregnancy. Abnormalities in the endometrium, such as decreased receptivity and disrupted endometrial shedding, can potentially affect embryo implantation and pregnancy maintenance, highlighting the vital role of the endometrium in reproduction. An overall summary of these findings is shown in Table 3.

**Table 3:** Roles of m6A protein machineries and biological mechanisms exerted in endometrium.

Regulator	Role	Mechanism	Authors [Ref.]
**METTL16/WTAP/ALKBH5/IGF2BP2**	The m6A levels in the uterus increase with the progression of pregnancy. However, in infertile patients, there is a dysregulation of m6A regulatory factors in the endometrium.	N/A	Zhao et al., 2021 ^50^
**METTL3**	Knocking down METTL3 affects the progression of pregnancy.	Knocking down METTL3 reduces the levels of CTGF mRNA, thereby affecting the proliferation of endometrial epithelial cells.	Sun et al., 2023 ^51^
	Downregulation of METTL3 expression leads to impaired endometrial receptivity and abnormal decidualization.	Depletion of METTL3 leads to enhanced mRNA stability of estrogen-responsive genes, such as Elf3 and Celsr2, but results in decreased expression levels of PR and its target genes in the endometrium, leading to insufficient progesterone responsiveness.	Wan et al., 2023 ^53^
	METTL3 is essential for *in vitro* decellularization of human endometrial stromal cells.	The loss of Mettl3 is accompanied by a significant decrease in PGR protein expression. Pgr mRNA is a direct target of m6A modification mediated by METTL3 and enhances PGR protein translation efficiency in a YTHDF1-dependent manner.	Zheng et al., 2023 ^54^
**METTL14**	Deficiency in Mettl14 leads to the failure of embryo implantation.	Deficiency in Mettl14 leads to abnormal upregulation of the uterine estrogen receptor α signaling and ERα phosphorylation, as well as abnormal activation of the innate immune pathway.	Kobayashi et al., 2023 ^52^

Zhao et al show that m^6^A levels in the endometrium increase as pregnancy progresses and that the localization of m^6^A regulatory factors in the uterus changes during pregnancy. In patients with infertility, these regulatory factors are dysregulated in the endometrium. Specifically, the mRNA levels of METTL16 and WTAP are down-regulated, whereas the mRNA levels of ALKBH5 and IGF2BP2 are abnormally up-regulated. Consequently, these changes lead to abnormalities in various immune-related pathways in women with infertility.^60^ Similarly, Sun et al report that METTL3 is highly expressed in the endometrial glandular epithelium from days 16–25 of pregnancy. It can be up-regulated by estrogen and progesterone in the goat uterus and primary endometrial epithelial cells. METTL3 knockdown leads to a decrease in connective tissue growth factor (CTGF) mRNA levels, affecting the proliferation of endometrial epithelial cells and thereby influencing pregnancy.^61^

Endometrial receptivity is closely associated with successful embryo implantation and is strongly affected by ovarian steroid hormones. Kobayashi et al report that mice lacking Mettl14 are infertile. Further studies reveal that estrogen receptor alpha (ERα) signaling and ERα phosphorylation are abnormally increased in Mettl14-deficient uteri. Additionally, abnormal activation of the innate immune pathway occurs, leading to embryo implantation failure.^62^ Similarly, in women with infertility associated with endometrial factors, METTL3 expression is significantly down-regulated in the uterus. The depletion of METTL3 results in increased mRNA stability of estrogen-responsive genes such as E74-like ETS transcription factor 3 (Elf3) and cadherin EGF LAG seven-pass G-type receptor 2 (Celsr2). However, after METTL3 knockout, the expression levels of progesterone receptor and its target genes in the endometrium decrease, indicating inadequate progesterone responsiveness. Wan et al conclude that METTL3 promotes endometrial receptivity and female fertility by balancing estrogen and progesterone signalling.^63^ Building upon these findings, Zheng et al further show that the loss of Mettl3 is accompanied by a significant reduction in progesterone receptor (PGR) protein expression. The Pgr mRNA is a direct target of the METTL3-mediated m^6^A modification and increases PGR protein translation in a YTHDF1-dependent manner. These findings ultimately demonstrate the necessity of METTL3 in the decidualization of human endometrial stromal cells *in vitro*.^64^

However, Xue et al report that methylation and METTL3 expression are significantly increased in the endometrium of patients with recurrent implantation failure. METTL3 can catalyze the methylation of the homeobox A10 (HOXA10) mRNA, leading to its degradation and subsequently causing a decrease in β3 integrin expression. Additionally, abnormal up-regulation of empty spiracles homeobox 2 (EMX2) occurs, which further affects embryo implantation.^65^ Both excessive and insufficient METTL3 levels can lead to changes in endometrial receptivity. However, the specific mechanisms underlying these changes require further investigation.

Based on current research, the m^6^A modification plays a critical role throughout the reproductive cycle by regulating key biological processes such as endometrial receptivity and decidualization. Notably, the endometrium comprises heterogeneous cell populations, including epithelial cells, vascular endothelial cells, and diverse immune cells. However, the specific cellular subtypes predominantly influenced by the m^6^A modification remain undefined, warranting focused investigation in future studies. Furthermore, the endometrium undergoes cyclic remodeling during the menstrual cycle, yet the potential fluctuations in m^6^A regulatory factors across these dynamic phases have not been systematically characterized. Elucidating such temporal variations in m^6^A modifiers could substantially advance our understanding of their functional mechanisms in endometrial physiology and pathology.

### m^6^A and the reproductive immune environment

The immune system plays a vital role in regulating and protecting the female reproductive process. However, abnormalities in its activation or dysfunction can potentially result in female infertility.^66^ Autoimmune reactions, anti-embryo responses, decreased immune regulatory function, and certain chronic inflammatory responses may lead to abnormal ovulation, decreased endometrial receptivity, difficulties in embryo implantation, and embryonic growth disorders, ultimately contributing to the occurrence of infertility. Multiple types of immune cells, including natural killer (NK) cells and T cells, actively contribute to and synchronize processes during pregnancy.^67^ However, currently, no specific research on how m^6^A regulates reproductive immunity has been published, but we can gain some insights from other studies. An overall summary of this topic is shown in Table 4.

**Table 4:** Roles of m6A protein machineries and biological mechanisms exerted in immune environment.

Regulator	Role	Mechanism	Authors [Ref.]
***NK cell***			
**YTHDF2**	YTHDF2 maintains the homeostasis and terminal maturation of NK cells and participates in regulating NK cell trafficking and Eomes.	YTHDF2 forms a positive feedback loop with STAT15-YTHDF5 to promote the effector function of NK cells.	Ma et al., 2021 ^60^
**METTL3**	Loss of METTL3 alters the homeostasis of NK cells and inhibits their function.	The protein expression of SHP-2 is reduced in METTL3-deficient NK cells, and decreased SHP-2 activity leads to a diminished response of NK cells to IL-15.	Song et al., 2021 ^61^
**FTO**	Defects in FTO lead to excessive activation of NK cells.	FTO negatively regulates IL-2/15-driven JAK/STAT signaling by increasing the mRNA stability of suppressor of cytokine signaling (SOCS) genes.	Kim et al., 2023 ^62^
***Treg cell***			
**METTL3**	Loss of Mettl3 leads to impaired suppressive function of Treg cells.	Loss of Mettl3 leads to increased levels of Socs mRNA, thereby targeting the IL-2-STAT5 signaling pathway, which tightly controls Treg cell function.	Tong et al., 2018 ^63^
**METTL14**	METTL14 reduces the inhibition of Treg cell proliferation.	N/A METTL14 reduces the influence on the SOCS family, thereby inhibiting the expansion of Treg cells.	Liu et al., 2022 ^64^
**ALKBH5/IGF2BP2**	ALKBH5 deficiency inhibits Treg cell recruitment.	The deficiency of ALKBH5, mediated by IGF2BP2, leads to enhanced stability of CCL28 mRNA, thereby inhibiting Treg recruitment.	Chen et al., 2023 ^65^
**YTHDF2**	The loss of YTHDF2 can lead to increased apoptosis of Treg cells and impaired suppressive function.	Elevated TNF signaling promotes the expression of YTHDF2, accelerating the degradation of m6A to regulate the NF-κB signaling pathway through modification of NF-κB negative regulatory factor encoding transcripts.	Zhang et al., 2023 ^66^

NK cells, the most abundant type of leukocyte during pregnancy, play crucial roles throughout the entire gestational period. In early pregnancy, NK cells account for 50%–70% of decidual lymphocytes. Although decidual NK cells have lower cytotoxicity, they release cytokines and chemokines that induce trophoblast invasion, tissue remodeling, embryo development, and placenta formation and contribute to immune defense. Premature activation of NK cells in late pregnancy can lead to the breakdown of maternal–fetal tolerance, subsequently causing preterm labor.^68^ Ma et al report that YTHDF2 maintains NK cell homeostasis and terminal maturation, regulating NK cell trafficking and eomesodermin (Eomes). Eomes plays key roles in embryonic development and immune modulation, making it indispensable for these important processes.^69^ YTHDF2 promotes NK cell effector functions and is required for IL-15-mediated NK cell survival and proliferation by forming a STAT5-YTHDF2 positive feedback loop. Transcriptome-wide screening identifies Tardbp as a YTHDF2-binding target in NK cells involved in regulating cell proliferation or survival.^70^

Additionally, Song et al reported a positive correlation between the protein expression levels of METTL3 and effector molecules in NK cells. The loss of METTL3 in NK cells alters their homeostasis and suppresses their function. Protein tyrosine phosphatase 2 (SHP-2) protein levels are reduced in METTL3-deficient NK cells, leading to a diminished NK cell response to IL-15.^71^ In addition to the influence of writers and readers, defects in the eraser FTO result in the excessive activation of NK cells. FTO negatively regulates IL-2/15-driven Janus kinase (JAK)/STAT signaling in NK cells by increasing the mRNA stability of suppressor of cytokine signaling (SOCS) genes, thereby modulating NK cell function.^72^ Overall, the m^6^A modification plays a pivotal role in orchestrating the involvement of NK cells in pregnancy by regulating their fate and function.

In the reproductive system, regulatory T cells (Tregs) perform an essential regulatory function, particularly in safeguarding the fetus against maternal immune responses. m^6^A methylation is significantly involved in preserving the suppressive function of Tregs.^73^ Tong et al reported that the loss of Mettl3 leads to an increase in Socs mRNA levels, thereby targeting the IL-2–STAT5 signaling pathway, which strictly controls Treg function. The SOCS family is also influenced by decreased expression of METTL14, which inhibits Treg expansion.^74^ As a demethylase, ALKBH5 deficiency, which is mediated by IGF2BP2, results in increased stability of the C–C motif chemokine ligand 28 (CCL28) mRNA, thereby inhibiting Treg recruitment.^75^ The loss of YTHDF2, an identifying protein, in Tregs significantly reduces tumor growth in mice, resulting in increased apoptosis of Tregs and leading to impaired suppressive functions.^76^

Studies have shown that the m^6^A modification plays a pivotal regulatory role in modulating the functions of NK cells and Tregs. However, current research on m^6^A-related immunology predominantly focuses on tumor biology, whereas investigations into reproductive immune regulation remain in their infancy. The immunologic processes during pregnancy constitute an extraordinarily complex regulatory network characterized by dynamic shifts in maternal immune environments from a proinflammatory state in early pregnancy to an immune-tolerant and anti-inflammatory state in mid-pregnancy and reverting to a proinflammatory state in late gestation. The potential functional roles of the m^6^A modification throughout these immunological phases represent a fascinating area of inquiry. Nevertheless, systematic explorations of m^6^A-mediated epigenetic regulation in pregnancy-related immune adaptation are currently limited, particularly regarding its mechanistic contributions to trophoblast–endometrial interactions, placental development, and maternal–fetal interface homeostasis. Further research is warranted to elucidate the spatiotemporal dynamics and molecular pathways of m^6^A modifications in orchestrating immune tolerance during healthy pregnancies and their perturbations in reproductive pathologies.

### m^6^A and the body's physiological state

**Figure 3:**
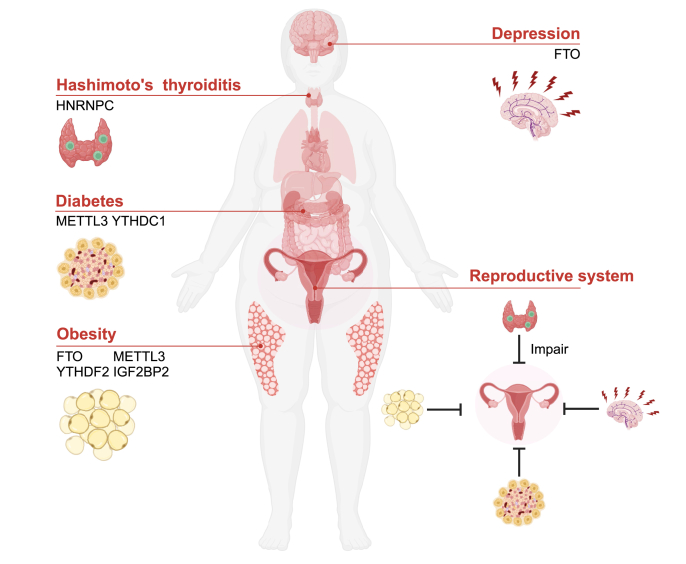
m^6^A and body physiological state. Pregnancy requires the support of a healthy physiological state, and dysregulation of m^6^A methylation can lead to abnormalities in the body's condition, including depression (FTO), Hashimoto's thyroiditis (hnRNPC), diabetes (METTL3, YTHDC1), obesity (FTO, YTHDF2, METTL3, IGF2BP2), thereby affecting conception. METTL3, methyltransferase 3; FTO, fat mass and obesity-associated protein; YTHDC1/2, YTH domain containing 1/2; hnRNPC, heterogeneous nuclear ribonucleoprotein C; IGF2BP2, insulin-like growth factor-2 mRNA-binding protein 2; YTHDF2, YTH domain family 2.

The physiological well-being of the body plays a crucial role in ensuring a healthy pregnancy. Factors that promote a healthy pregnancy include weight management, continued management of chronic diseases, and improved psychological well-being. Women who are overweight or underweight encounter certain obstacles to conception; nutritional deficits or an underweight status damage mainly the hypothalamic–pituitary–gonadal axis, leading to hypothalamic anovulation and affecting fertility.^77^ The impact of obesity on fertility is complex. In addition to interfering with the hypothalamic–pituitary–ovarian axis, excess adipose tissue also leads to hormonal disruption and abnormal levels of inflammation within the body. Obesity is often associated with a series of chronic metabolic diseases, such as diabetes, hypertension, and hyperlipidemia, which pose major challenges to conception.^78^ In recent years, multiple studies have shown correlations between obesity and increased incidences of depression and anxiety, suggesting that obesity is a key risk factor for the development of these disorders.^79^ Therefore, appropriate weight management before pregnancy is particularly crucial. The overall summary of this part is shown in Figure 3 and Table 5.

**Table 5:** Roles of m6A protein machineries and biological mechanisms exerted in organism status.

Regulator	Role	Mechanism	Authors [Ref.]
***Obesity***			
FTO	FTO regulates the process of adipogenesis.	FTO controls adipogenesis by regulating the m6A levels and modulating the exon splicing of the adipogenic regulator RUNX1T1.	Zhao et al., 2014 ^72^
FTO/YTHDF2	FTO/YTHDF2 regulate adipogenesis.	In the case of FTO silencing, the m6A-modified Atg5 and Atg7, with higher levels, can be recognized by YTHDF2, leading to reduced autophagy and adipogenesis	Wang et al., 2020 ^73^
METTL3/IGF2BP2	Deficiency of METTL3 in mature beige adipocytes can lead to inhibition of adipocyte glycolytic capacity and thermogenic capacity.	METTL3 and IGF2BP2 can control the mRNA stability of key glycolytic genes in beige adipocytes.	Li et al., 2023 ^74^
METTL3	Silencing of Mettl3 can severely inhibit the maturation of brown adipocytes.	Reduced m6A modification and decreased expression of Prdm16, Pparg, and Ucp1 transcripts. This results in a significant decrease in brown fat-mediated adaptive thermogenesis and promotes obesity and systemic insulin resistance induced by a high-fat diet.	Wang et al., 2020 ^75^
***Diabetes***			
METTL3	The specific loss of Mettl3 in pancreatic islet β-cells leads to β-cell dysfunction and high blood sugar levels.	Reduced m6A modification leads to a decrease in the expression of insulin secretion-related genes.	Li et al., 2021 ^77^
YTHDC1	YTHDC1 is downregulated in pancreatic β-cells of type 2 diabetes, resulting in a decrease in insulin levels.	YTHDC1 leads to a decrease in the expression of beta-cell-specific transcription factors and insulin secretion-related genes.	Li et al., 2023 ^78^
***Depression***			
FTO	Inhibiting the expression of FTO can lead to the development of depression-like behavior in adult mice.	ADRB2 mRNA is a target of FTO, and stimulation of ADRB2 can restore depression-like behavior in FTO-deficient mice.	Liu et al., 2021 ^79^
***Hashimoto's thyroiditis***			
HNRNPC	Abnormal expression of hnRNPC mediates the development of Hashimoto's thyroiditis.	hnRNPC promotes ATF4 transcriptional activity, synthesis, and translation through m6A modification. Increased ATF4 expression triggers endoplasmic reticulum stress signaling, leading to apoptosis and necroptosis of thyroid follicular epithelial cells.	Mo et al., 2021 ^82^

Numerous studies have provided evidence to support the significant involvement of the m^6^A modification in the initiation and progression of obesity. METTL3/METTL14 and FTO are involved in the accumulation of lipids in various cells by influencing lipid breakdown and synthesis. YTHDF proteins recognize m^6^A methylation sites on RNA and regulate the translation of target genes.^80^ FTO controls the exon splicing of the fat generation regulator, Runt-related transcription factor 1 (RUNX1) partner transcriptional co-repressor 1 (RUNX1T1), by modulating m^6^A levels, thereby regulating the process of adipocyte differentiation.^81^^,^^82^ Additionally, FTO directly targets autophagy-related 5 (Atg5) and Atg7 and mediates their expression in an m^6^A-dependent manner. Further research reveals that Atg5 and Atg7 are targets of YTHDF2. In the absence of FTO, YTHDF2 can recognize increased levels of m^6^A-modified Atg5 and Atg7, leading to mRNA degradation and decreased protein expression, thus alleviating autophagy and fat production.^83^ In addition to FTO, METTL3 regulates fat metabolism, and METTL3 deficiency in mature beige adipocytes can lead to the inhibition of the glycogenolytic capacity and thermogenesis. Both METTL3 and IGF2BP2 control the mRNA stability of key glycogenolytic genes in beige adipocytes.^84^ Furthermore, METTL3 is involved in the regulation of brown adipocytes. Mettl3 silencing in brown adipocytes substantially inhibits their maturation and reduces the level of the m^6^A modification as well as the expression of PR domain-containing 16 (Prdm16), peroxisome proliferator-activated receptor gamma (Pparg), and uncoupling protein 1 (Ucp1) transcripts *in vivo*. These changes result in a significant reduction in adaptive thermogenesis mediated by brown adipose tissue and promote obesity and systemic insulin resistance induced by high-fat diet consumption.^85^

In addition to obesity, m^6^A methylation also significantly influences various chronic metabolic disorders. Extensive research has shown that the demethylase FTO is one of the greatest genetic risk factors for type 2 diabetes.^86^ METTL3 regulates β-cell function and diabetes. Under inflammatory and oxidative stress conditions, METTL3 is down-regulated, and β-cell-specific Mettl3 deficiency results in β-cell dysfunction and hyperglycemia, possibly due to reduced levels of the m^6^A modification, which leads to decreased expression of insulin secretion-related genes.^87^ YTHDC1 is down-regulated in β-cells in individuals with type 2 diabetes, leading to decreased insulin levels that possibly result from the reduced expression of β-cell-specific transcription factors and insulin secretion-related genes caused by YTHDC1.^88^ Moreover, research has revealed a close association between the down-regulation of FTO expression and depression. The inhibition of FTO expression in the hippocampus leads to the development of depression-like behaviors in adult mice, whereas the overexpression of FTO results in the restoration of depression-like phenotypes. Further investigations have shown that the adrenoceptor beta 2 (ADRB2) mRNA is a target of FTO and that the stimulation of ADRB2 can ameliorate depression-like behaviors in FTO-deficient mice.^89^

In addition to metabolic disorders and depression, systemic autoimmune diseases can also pose challenges to pregnancy.^90^ Hashimoto's thyroiditis is an autoimmune thyroid disease that can lead to reduced fertility, an increased risk of miscarriage, and pregnancy-related complications such as premature birth and low birth weight.^91^ Recent research has shown that hnRNPC promotes activating transcription factor 4 (ATF4) transcriptional activity, synthesis, and translation through the m^6^A modification. Increased ATF4 expression triggers endoplasmic reticulum stress signaling, leading to the apoptosis and necroptosis of thyroid follicular epithelial cells and thereby mediating the development of Hashimoto's thyroiditis. However, targeting hnRNPC significantly reduces the death of thyroid follicular epithelial cells to ameliorate disease progression, indicating the involvement of m^6^A methylation in thyroid diseases.^92^

In summary, m^6^A methylation plays a role in regulating organismal states, and many m^6^A-related genes, especially those related to the demethylase FTO, play important roles in obesity, chronic diseases, and depression, thereby affecting female fertility. Successful pregnancy requires the close coordination of the reproductive system and relies on a well-functioning organismal state. The systemic effects of m^6^A should be a key consideration during pregnancy. Additionally, further research should investigate whether conditions such as obesity can impact the m^6^A modification in the reproductive system, which could be the focus of future studies.

### Summary of the role of m^6^A in reproductive physiology

m^6^A methylation plays multidimensional regulatory roles in reproductive physiology, with its molecular mechanisms spanning critical processes, including oocyte development, GC function, endometrial receptivity, reproductive immune regulation, and the physiological state. Current research limitations include the following: i) insufficient elucidation of the downstream pathways and molecular alterations mediated by m^6^A modifications, despite the observed changes in regulatory factors and associated phenotypes; ii) oversimplification of the dynamic and reversible nature of the m^6^A modification, which involves complex interactions among multiple regulatory factors, whereas existing studies predominantly focus on individual components; and iii) fragmented investigations of long-term cyclical processes in reproduction and pregnancy, such as oocyte maturation (from primordial to mature stages), phased endometrial remodeling (proliferative to secretory phases), and dynamic immune state transitions during gestation, with current research often isolating specific phases rather than adopting comprehensive temporal perspectives. Future research directions necessitate establishing cross-scale analytical frameworks that integrate organoid models with multiomics technologies while strengthening validation through the analysis of human reproductive tissue samples to facilitate the clinical translation of m^6^A regulatory mechanisms.

### The roles of m^6^A methylation in different female reproductive diseases

**Figure 4:**
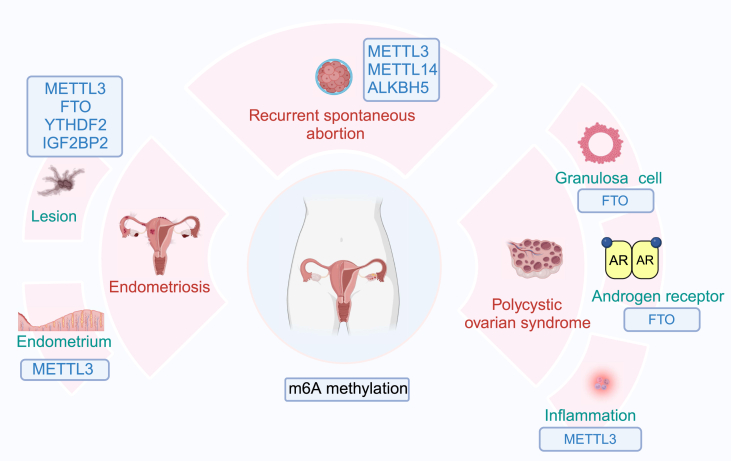
The role of m^6^A methylation in different female reproductive diseases. m^6^A modification plays a key role in multiple female reproductive diseases, such as endometriosis (METTL3, FTO, YTHDF2, IGF2BP2), polycystic ovary syndrome (FTO, METTL3), and recurrent spontaneous abortion (METTL3, METTL14, ALKBH5). METTL3/14, methyltransferase 3/14; ALKBH5, ALKB homologue 5; FTO, fat mass and obesity-associated protein; YTHDC2, YTH domain containing 2; IGF2BP2, insulin-like growth factor-2 mRNA-binding protein 2; YTHDF2, YTH domain family 2.

In addition to the various stages of conception mentioned above, the m^6^A modification plays a key role in multiple female reproductive diseases, such as endometriosis (EM), polycystic ovary syndrome (PCOS), and recurrent spontaneous abortion (RSA). These diseases can also affect a woman's ability to conceive. Therefore, exploring the differences in m^6^A levels and changes in downstream mechanisms in these female reproductive diseases may lead to new treatment targets. The details can be seen in Figure 4 and Table 6.

**Table 6:** Roles of m6A protein machineries and biological mechanisms exerted in female reproductive system diseases.

Regulator	Mechanism	Functional classification	Authors [Ref]
***Endometriosis***			
METTL3	Weaken the maturation of pri-miR126 mediated by DGCR8.	Promote the proliferation, invasion and migration of endometrial stromal cells.	Li et al., 2021 ^85^
	Regulate m6A modification of SIRT1 mRNA by the identifying function of YTHDF2 and target SIRT1/FoxO3a signaling to enhance cellular senescence.	Inhibit the migration, invasion and proliferation of endometrial stromal cells.	Wang et al., 2023 ^88^
	Inhibit the stability and expression of WIF1 mRNA.	Promote the proliferation, invasion and migration of endometrial stromal cells.	Zhang, 2023 ^86^
	Mediate m6A modification of Trib1 to activate ERK/STAT3 signaling pathway and induce M2 macrophage polarization.	Promote the progression of EM ectopic lesions.	Gou et al., 2022 ^87^
	Regulate YTHDF2-mediated degradation of FOXO1 mRNA.	Impair the decidualization of endometrial stromal cells.	Li et al., 2023 ^92^
IGF2BP2	Stabilize mRNA levels of MEIS2 and GATA6.	Promote the proliferation, invasion and migration of endometrial stromal cells.	Zhao et al., 2022 ^90^
FTO	Reduce m6A modification of ATG5 to increase the level of autophagy and inhibit the expression of PKM2.	Inhibit the level of glycolysis, proliferation and migration of endometrial stromal cells.	Wang et al., 2022 ^91^
***PCOS***			
METTL3	Upregulate the levels of NLRP3 protein and inflammatory cytokines (IL-6 and TNF-α) by increasing the level of m6A methylation and mRNA expression of FOSL2.	Impair ovarian function and induce inflammation.	Liu et al., 2023 ^49^
FTO	Promote the expression of FLOT2 by decreasing the m6A level of FLOT2 mRNA and increasing its stability.	Promote the proliferation of GCs, hinder apoptosis of GCs and induce IR.	Zhou et al., 2022 ^99^
	Enhance the expression of steroidogenic enzymes and AR and increase AR/AKT signaling.	Lead to hyperandrogenism.	Jing et al., 2023 ^100^
***Recurrent spontaneous abortion***			
METTL3	Regulate the stability of ZBTB4 mRNA and upregulate the expression of ZBTB4 by mediating m6A modification.	Attenuate the proliferation and migration of trophoblast cells in the villous tissue of RSA placenta.	Huang et al., 2022 ^105^
	Enhance the stability and expression of lncHZ09 by upregulating the m6A modification of its RNA.	Inhibit the migration and invasion of trophoblast cells and induce RSA.	Dai et al., 2023 ^109^
	Enhance the m6A modification and stability of NLRP3 mRNA mediated by METTL3.	Promote pyroptosis of trophoblast cells.	Wang et al., 2023 ^110^
ALKBH5	Lead to the insufficient secretion of VEGF.	Impair the recruitment and M2 differentiation of macrophage, ultimately leading to the occurrence of RSA.	Zhao et al., 2022 ^106^
	Shorten the half-life period of CYR61 mRNA by regulating the level of m6A methylation and decrease the expression of steady-state CYR61 mRNA.	Inhibit the invasion of trophoblast.	Li et al., 2019 ^104^
	Enhance the expression of SMAD1/5 by eliminating m6A modification, thereby up-regulating the levels of MMP9 and ITGA1.	Promote the activity, migration and invasion of trophoblast cells.	Zheng et al., 2022 ^107^
METTL14	Enhance the RNA stability and expression of lnc-HZ01 by catalyzing m6A methylation.	Inhibit the proliferation of trophoblast cells and induce the occurrence of RSA.	Xu et al., 2021 ^108^

### m^6^A methylation and EM

EM is a chronic inflammatory disease characterized by the presence of endometrial glands and stroma outside the uterine cavity covering the mucosa and myometrium.^93^ It is characterized by chronic pelvic pain and infertility, and seriously affects the physical and mental health of women of childbearing age.^94^ Increasing evidence shows that m^6^A methylation plays an important role in EM and related infertility and that further study of m^6^A methylation may lead to the identification of diagnostic biomarkers and therapeutic targets.

The METTL3-mediated m^6^A modification is the most studied in EM. A down-regulated expression level of METTL3 in EM-overexpressing endometrial tissues and stromal cells can lead to a decreased m^6^A modification level and weakened DiGeorge syndrome critical region 8 (DGCR8)-mediated pri-miR126 maturation in an m^6^A-dependent manner, thereby promoting the proliferation, invasion, and migration of endometrial stromal cells and ultimately promoting the progression of EM.^95^ miR-21-5p can inhibit the expression of METTL3 and the subsequent METTL3-mediated m^6^A modification, thereby reducing mRNA stability; inhibiting the expression of WNT inhibitory factor 1 (WIF1); and ultimately promoting the proliferation, invasion, and migration of endometrial stromal cells.^96^ In addition, another study reveals that lactic acid can increase the METTL3-mediated m^6^A modification of Tribbles pseudokinase 1 (Trib1), thereby activating the extracellular signal-regulated kinase (ERK)/STAT3 signaling pathway to induce M2 macrophage polarization and ultimately promote the progression of EM-related ectopic lesions.^97^ On the other hand, another study showed that METTL3 can interact with YTHDF2 to regulate the m^6^A modification of the sirtuin 1 (SIRT1) mRNA and target SIRT1/forkhead transcription factor 3a (FoxO3a) signaling to increase cellular senescence, ultimately preventing the migration, invasion, and proliferation of endometrial stromal cells and inhibiting the biological progression of EM.^98^ In summary, the regulatory effect of METTL3 on the progression of EM lesions may be bidirectional and closely related to its regulating downstream mRNAs. However, further studies are needed to determine which mRNAs play a dominant role in this process.

In addition to METTL3, several other m^6^A regulatory factors play key roles. METTL14 expression is significantly down-regulated in the ectopic endometria of EM patients. Moreover, METTL14 knockdown can increase the proliferation and invasion of endometrial stromal cells.^99^ Zhao et al show that IGF2BP2 and its related target genes, myeloid ecotropic viral integration site 1 homolog 2 (MEIS2) and GATA binding protein 6 (GATA6), are highly expressed in EM.^100^ IGF2BP2 can promote the proliferation, migration, and invasion of endometrial stromal cells by stabilizing the MEIS2 and GATA6 mRNAs. The expression of the demethylase FTO is significantly down-regulated in ectopic endometrial tissue and stromal cells in patients with EM, and the overexpression of FTO can increase the level of autophagy by targeting ATG5 to reduce the level of the m^6^A modification, thereby down-regulating the expression of PKM2 to inhibit glycolysis, proliferation, and migration in endometrial stromal cells.^101^

In addition to affecting EM itself, m^6^A plays a role in EM-related infertility. An analysis of the endometria of infertile EM patients reveals that the levels of METTL3 and m^6^A are significantly increased, whereas the expression of FOXO1 is decreased.^102^ Moreover, METTL3 overexpression inhibits decidual marker expression and embryo implantation. Further studies reveal that the METTL3-mediated m^6^A modification impairs the decidualization of endometrial stromal cells by regulating the YTHDF2-mediated degradation of the FOXO1 mRNA, thereby leading to EM-related infertility.

m^6^A methylation plays a pivotal role in EM. The METTL3-mediated modification reduces m^6^A levels, thereby promoting the proliferation, migration, and invasion of endometrial stromal cells. Other m^6^A regulators, including METTL14, IGF2BP2, and FTO, are also implicated in EM pathogenesis. Mechanistically, m^6^A influences decidualization and embryo implantation by regulating FOXO1 mRNA degradation, thereby contributing to EM-associated infertility. However, current research has notable limitations: investigations into regulators such as METTL14 and FTO remain confined to phenotypic observations without a systematic exploration of specific molecular targets or pathways. Furthermore, existing studies have failed to delineate the unique dynamic alterations in m^6^A modification patterns between EM lesions and normal endometrial tissues. These gaps underscore the necessity of integrating multiomics analyses with functional validation experiments to comprehensively elucidate the molecular network of m^6^A in EM. Future studies should prioritize the stepwise elucidation of the distinct roles of m^6^A in ectopic lesions versus eutopic endometrium, particularly focusing on specific spatiotemporal regulatory mechanisms and their clinical translational potential.

### m^6^A methylation and PCOS

PCOS is a chronic low-grade inflammatory disease caused by multiple factors, including genetics, endogenous factors, endocrine regulation, metabolic disorders, and the environment, and is usually accompanied by obesity and insulin resistance.^103^ Treatment strategies for PCOS include a combination of lifestyle optimization and medical intervention. However, due to the complexities of its diagnosis and subsequent treatment, the efficacy of therapy is unsatisfactory, highlighting the need for further research on the pathogenesis of PCOS.^104^ Many studies have shown that m^6^A regulatory factors play important roles in the biological process of PCOS.^105^^,^^106^

Ovarian GC dysfunction is an important pathophysiological feature of PCOS. FOXO3 is highly expressed in GCs and is closely related to increased apoptosis.^107^^,^^108^ However, the mechanism underlying the regulation of FOXO3 expression remains unclear. Given this information, researchers, including Zhang et al, have shown a decrease in the m^6^A modification of the FOXO3 mRNA in luteinized GCs after controlled ovarian hyperstimulation in PCOS patients,^106^ which disrupts the regulation of FOXO3 expression in GCs, preliminarily indicating that changes in the m^6^A modification are responsible for disorders of certain key genes in patients with PCOS. In addition, abnormal expression of FTO, which is also closely related to GC dysfunction, can increase the risks of PCOS and insulin resistance. Another study reveals that the expression level of flotillin-2 (FLOT2) in the GCs of PCOS patients is significantly higher than that in the GCs of women with a normal ovarian reserve,^109^ and the bioinformatics analysis reveals that FLOT2 is a downstream target of FTO. The overexpression of FTO can promote FLOT2 expression by decreasing the m^6^A level on the FLOT2 mRNA in GCs and increasing its stability, thereby promoting cell proliferation, hindering apoptosis, and inducing insulin resistance. Therefore, the FTO/FLOT2 axis may play an important role in the pathophysiology of PCOS.

In addition to regulating FLOT2 in GCs, FTO is closely related to hyperandrogenism in PCOS patients. Jing et al show that FTO, which is significantly up-regulated in PCOS patients, is positively correlated with androgen levels and negatively correlated with m^6^A levels.^110^ Inhibiting FTO can affect the expression of steroidogenic enzymes and androgen receptor (AR) and ameliorate hyperandrogenism in patients with PCOS by decreasing AR/AKT signaling, which provides a new theoretical basis for the clinical diagnosis and treatment of PCOS.

The gut microbiome can also influence the m^6^A modification level in host tissue cells. Liu et al have revealed a new possible pathogenesis of PCOS through a correlation analysis between the gut microbiota and PCOS.^59^ The results show that an abnormal gut microbiota in obese PCOS patients can lower the level of butyric acid and may up-regulate the expression of METTL3 to increase the levels of the m^6^A modification and inflammation. Mechanistically, butyric acid can decrease the level of m^6^A methylation and FOSL2 mRNA expression by inhibiting the expression of METTL3, thereby leading to a decrease in the levels of the NLRP3 protein and inflammatory cytokines such as IL-6 and TNF-α. Therefore, butyric acid supplementation can improve ovarian function and attenuate inflammation by inhibiting the METTL3/FOSL2/NLRP3 pathway, and this approach is expected to become a new method for the treatment of PCOS.

m^6^A methylation influences key processes, such as ovarian GC dysfunction and hyperandrogenism, thereby affecting the progression of PCOS. However, PCOS patients exhibit significant clinical heterogeneity, including distinctions between obese and nonobese individuals and between insulin-resistant and nonresistant subgroups. The specific regulatory patterns of the m^6^A modification across these subtypes remain unclear. Additionally, large-scale cohort studies on PCOS to validate the molecular associations between m^6^A-related genetic variations and specific phenotypes, such as the relationship between FTO gene variants and ovulatory dysfunction, are lacking. Furthermore, research on the regulatory effects of critical environmental factors, such as dietary habits and metabolic status, on the m^6^A modification in individuals with PCOS remains insufficient. These knowledge gaps hinder the establishment of targeted therapeutic strategies and the development of personalized interventions based on m^6^A dynamics.

### m^6^A methylation and RSA

The occurrence of two or more consecutive abortions is referred to as RSA, which is a common complication of early pregnancy. Although many studies have shown that risk factors such as chromosomal abnormalities, abnormalities in the uterine anatomy, and endocrine abnormalities may lead to early pregnancy loss, the clinical causes of approximately half of RSAs remain unclear.^111^ An increasing number of studies have shown that many types of dysfunction, such as the inhibition of the proliferation, migration, and invasion of human trophoblast cells, are closely related to adverse pregnancy outcomes, such as RSA.^112^^,^^113^ In addition, the abnormal m^6^A modification of RNA may be related to the occurrence of trophoblast dysfunction and trophoblast-related adverse pregnancy outcomes.^114^ To date, studies on the role of the m^6^A modification in RSA have focused mainly on villus tissue and trophoblast cells.

Huang et al reported that the expression of METTL3 in the placental villous tissue of RSA patients was significantly decreased.^115^ Mechanistically, METTL3 can regulate the stability of the zinc finger and BTB domain containing 4 (ZBTB4) mRNA and up-regulate the expression of ZBTB4 by mediating the m^6^A modification, thereby attenuating the proliferation and migration of trophoblast cells in the villous tissue of RSA placentas. Among the many m^6^A regulators, ALKBH5 is the most studied among the RSA-related m^6^A modifications. Zhao et al report that one of the characteristics of RSA is high expression of ALKBH5 in stromal cells and subsequent impaired macrophage differentiation, revealing a new theory of the interaction between these two cell types.^116^ The overexpression of ALKBH5 in stromal cells can lead to insufficient secretion of vascular endothelial growth factor (VEGF), which may impair the recruitment and M2 differentiation of macrophages, ultimately leading to the occurrence of RSA. A study of villous tissue reveals that the significantly higher expression of ALKBH5 in the placental villous tissue of RSA patients can lead to a significant reduction in the level of m^6^A methylation.^114^ In contrast, a reduction in ALKBH5 expression can extend the half-life of the cystein-rich protein 61 (CYR61) mRNA by regulating the level of m^6^A methylation and promoting the steady-state expression of the CYR61 mRNA, thereby promoting trophoblast invasion. However, another study reported opposite results. A study by Zheng et al revealed that the expression of ALKBH5 in the extravillous trophoblasts of RSA patients was significantly decreased, leading to the inhibition of trophoblast invasion and the occurrence of RSA.^117^ However, the expression of ALKBH5, which is translocated from the nucleus to the cytoplasm, was significantly increased after hypoxia treatment. Moreover, ALKBH5 can increase the expression of SMAD1/5 by eliminating the m^6^A modification, thereby increasing the expression of matrix metalloproteinase 9 (MMP9) and integrin α1 (ITGA1) and ultimately promoting the activity, migration, and invasion of trophoblast cells. The reason for the inconsistencies between these two studies may be that these cells were cultured in different environments under normoxic and hypoxic conditions, which led to different activation states of the hypoxia-inducible factor 1-alpha (HIF-1α) signaling pathway, thereby affecting trophoblast activity in early pregnancy.

Benzo(a)pyrene (BaP) and its final metabolite BPDE (benzo(a)pyrene-7,8-dihydrodiol-9,10-epoxide) in the environment are typical persistent organic pollutants and endocrine disruptors to which trophoblast cells are very sensitive. Exposure to BaP/BPDE may impair the function of trophoblast cells through lncRNAs modified by m^6^A, thereby inducing the occurrence of RSA. Several studies have shown that novel lncRNAs, including lnc-HZ01, lnc-HZ09, and lnc-HZ14, are significantly highly expressed in the villous tissue of patients with RSA and trophoblast cells exposed to BPDE.^118^, ^119^, ^120^ Among them, lnc-HZ01 can increase the mRNA transcription and protein stability of MAX dimerization protein 1 (MXD1) by up-regulating its transcription factor c-JUN (activating protein-1 transcription factor subunit) and deubiquitinase ubiquitin-specific protease 36 (USP36).^118^ In addition, MXD1 can promote the transcription of METTL14, thereby increasing the RNA stability and expression of lnc-HZ01 by catalyzing m^6^A methylation. Therefore, lnc-HZ01 and MXD1 can form a significantly up-regulated positive self-feedback loop in RSA trophoblast tissue, which can inhibit the proliferation of trophoblast cells and induce the occurrence of RSA. Dai et al show that exposure to BPDE can increase the expression of METTL3, thereby increasing the stability and expression of lncHZ09 by increasing the m^6^A modification of its RNA and ultimately weakening the SP1-mediated transcription and stability of the phospholipase D1 (PLD1) mRNA.^119^ Moreover, lnc-HZ09 inhibits the migration and invasion of trophoblast cells and subsequently induces RSA by inhibiting the PLD1/Rac family small GTPase 1 (RAC1)/cell division cycle 42 (CDC42) pathway. Wang et al show that exposure to BaP/BPDE can induce pyroptosis in trophoblast cells by up-regulating the lnc-HZ14/Z-DNA binding protein 1 (ZBP1)/NLRP3 axis, ultimately leading to RSA.^120^ Mechanistically, lnc-HZ14 promotes the transcription of the ZBP1 mRNA via interferon regulatory factor 1 (IRF1), enhances the m^6^A modification and stability of the NLRP3 mRNA through a mechanism mediated by METTL3, and ultimately promotes pyroptosis in trophoblast cells. These findings provide new insights into the destruction of trophoblast cells induced by BaP/BPDE and the pathogenesis of unexplained RSA.

Alterations in m^6^A regulation, including decreased METTL3 expression and altered ALKBH5 expression, can impair trophoblast cell proliferation, migration, and invasion, potentially contributing to RSA. Environmental exposures such as BaP/BPDE may exacerbate trophoblast dysfunction through m^6^A-modified lncRNAs that interfere with cellular functions. Key lncRNAs, including lnc-HZ01, lnc-HZ09, and lnc-HZ14, are up-regulated in individuals with RSA and promote trophoblast dysfunction by destabilizing cellular homeostasis or inducing cell death. While these findings position m^6^A as a potential therapeutic target for RSA, critical knowledge gaps persist. Specifically, the direct pathogenic roles and molecular mechanisms linking reduced m^6^A methylation levels to impaired trophoblast invasiveness remain incompletely understood. Furthermore, current research lacks cell-type-specific m^6^A mapping studies, particularly those focused on trophoblast cells at the maternal–fetal interface, resulting in overly generalized conclusions about the roles of m^6^A-mediated regulatory networks in RSA pathogenesis.

### Summary of the roles of m^6^A methylation in different female reproductive diseases

The investigation of m^6^A regulation in reproductive disorders has revealed several persistent challenges. First, clinical translation remains challenging, as most studies are confined to basic experiments or small-scale observational investigations. Large-scale cohort validation and standardized detection methods are notably absent, but the sensitivity, specificity, and generalizability of m^6^A regulatory factors as potential biomarkers across disease subtypes remain inadequately validated. Second, the insufficient exploration of m^6^A dynamics and reversibility under pathological conditions limits a comprehensive understanding. Although m^6^A modifications exhibit spatiotemporal dynamics, current research predominantly focuses on static differences. For example, EM involves distinct disease stages, and RSA encompasses gestational phase transitions; however, longitudinal analyses of m^6^A modification dynamics during these processes are lacking. Furthermore, therapeutic development lags behind mechanistic research. Despite accumulating evidence of the pathological roles of m^6^A, targeted drug discovery and clinical translation efforts remain critically underdeveloped, creating substantial barriers to practical applications.

## Discussion

In recent years, with the widespread application of high-throughput sequencing technologies and highly specific antibodies targeting m^6^A, m^6^A methylation has been recognized to play an increasingly important role in promoting RNA sorting and regulation within cells. This process facilitates organized cellular metabolism and functional regulation while enhancing the precise control of gene expression.^121^ The female reproductive process is a complex physiological phenomenon involving multiple factors, including oocyte development, GC function, endometrial receptivity, the immune system, and overall biological conditions. This article provides a comprehensive summary of the roles of m^6^A methylation across various aspects of female reproductive system diseases, aiming to analyze the impact of imbalanced m^6^A methylation on female infertility from a systemic and holistic perspective. These findings may provide valuable insights for advancing clinical treatment strategies.

However, some common issues still need to be addressed in current studies. First, most studies rely on bioinformatics analyses to study the expression profiles of m^6^A regulators and identify the characteristics of m^6^A regulators in specific diseases, and these studies often follow repetitive and formulaic approaches that provide limited research value. Second, few reports are available on the molecular mechanisms of m^6^A methylation and its regulators, including site-specific modifications and precise regulatory pathways, which are only satisfactory for a phenotypic exploration. In addition, studies of targeted agonists or inhibitors of these m^6^A regulators are lacking. Only a few reports on inhibitors of METTL3, FTO, and ALKBH5 have been published, all of which are focused on the field of oncology, and no study has been reported on women's diseases. Finally, most studies have focused on *in vitro* experiments with little *in vivo* validation and a lack of specific clinical applications, which severely hampers translational progress. Addressing these challenges will deepen our understanding of the intrinsic role of m^6^A methylation in the female reproductive system while providing more validated insights into disease treatment strategies.

Considering these issues, future research should fully explore changes in the upstream and downstream pathway involved in m^6^A regulation by combining more advanced multimodal genomics technologies, develop m^6^A-regulated drugs that are more suitable for treating women's diseases through better *in vivo* experimental studies, and then validate whether these therapies can achieve the expected therapeutic effects through multi-center and large-scale studies. The dynamic changes in m^6^A levels and its involvement in the whole process of reproduction indicate that the regulation of m^6^A must be individualized, specific, and continuously adjusted according to the stage of the patient, which may be a major difficulty in the clinical application of m^6^A; however, with the continuous progress of this research, a solution can certainly be found.

## Conclusions

In summary, m^6^A methylation serves as a dynamic and versatile regulatory mechanism that profoundly influences female reproductive physiology and pathology. This review highlights its indispensable roles in oocyte maturation, GC dynamics, endometrial receptivity, immune homeostasis, and systemic adaptations during pregnancy. The dysregulation of m^6^A modifications disrupts critical gene networks, contributing to infertility-associated conditions such as endometriosis, polycystic ovary syndrome, and recurrent miscarriage. Key enzymes such as METTL3, FTO, and ALKBH5 play dual roles in disease progression, underscoring the complexity of m^6^A-mediated regulation. Notably, m^6^A modifiers have strong potential as diagnostic biomarkers and therapeutic targets. However, challenges remain, including limited clinical validation, unresolved mechanistic details of specific m^6^A sites, and an insufficient exploration of m^6^A–immune interactions in reproductive contexts. Future research should prioritize translational studies to harness m^6^A pathways for clinical interventions, alongside mechanistic investigations into cell-type-specific roles and crosstalk with epigenetic or metabolic networks. Addressing these gaps will advance our understanding of m^6^A in reproductive health and pave the way for innovative strategies to combat female infertility, a globally significant medical issue.

## CRediT authorship contribution statement

**Jie Ding:** Writing – original draft, Methodology, Conceptualization. **Yalun He:** Data curation. **Yangshuo Li:** Visualization. **Shuai Sun:** Software. **Wen Cheng:** Supervision. **Jiami Huang:** Validation, Software, Methodology. **Chaoqin Yu:** Writing – review & editing.

## Funding

This work was supported by grants from the Medical Innovation Research Project of Science and Technology Innovation Action Plan of Shanghai Science and Technology Commission of China (No. 20Z21900405) and Shanghai Famous Chinese Medicine Academic Experience Research Studio (China) (No. SHGZS-202240).

## Conflict of interests

No potential conflict of interests was disclosed.

## References

[1] Peixoto P., Cartron P.F., Serandour A.A., Hervouet E. (2020). From 1957 to nowadays: a brief history of epigenetics. Int J Mol Sci.

[2] Cui L., Ma R., Cai J. (2022). RNA modifications: importance in immune cell biology and related diseases. Signal Transduct Targeted Ther.

[3] Wang H., Han J., Zhang X.A. (2025). Interplay of m6A RNA methylation and gut microbiota in modulating gut injury. Gut Microbes.

[4] Meyer K.D., Saletore Y., Zumbo P., Elemento O., Mason C.E., Jaffrey S.R. (2012). Comprehensive analysis of mRNA methylation reveals enrichment in 3' UTRs and near stop codons. Cell.

[5] An Y., Duan H. (2022). The role of m6A RNA methylation in cancer metabolism. Mol Cancer.

[6] Yue Y., Liu J., He C. (2015). RNA N6-methyladenosine methylation in post-transcriptional gene expression regulation. Genes Dev.

[7] Wei W., Ji X., Guo X., Ji S. (2017). Regulatory role of N6-methyladenosine (m^6^A) methylation in RNA processing and human diseases. J Cell Biochem.

[8] Zhang H., Shi X., Huang T. (2020). Dynamic landscape and evolution of m6A methylation in human. Nucleic Acids Res.

[9] Zhang W., Zhang S., Dong C. (2022). A bibliometric analysis of RNA methylation in diabetes mellitus and its complications from 2002 to 2022. Front Endocrinol.

[10] Jia G., Fu Y., Zhao X. (2011). N6-methyladenosine in nuclear RNA is a major substrate of the obesity-associated FTO. Nat Chem Biol.

[11] Jiang X., Liu B., Nie Z. (2021). The role of m6A modification in the biological functions and diseases. Signal Transduct Targeted Ther.

[12] Sharma R., Biedenharn K.R., Fedor J.M., Agarwal A. (2013). Lifestyle factors and reproductive health: taking control of your fertility. Reprod Biol Endocrinol.

[13] Roundtree I.A., Evans M.E., Pan T., He C. (2017). Dynamic RNA modifications in gene expression regulation. Cell.

[14] van Tran N., Ernst F.G.M., Hawley B.R. (2019). The human 18S rRNA m6A methyltransferase METTL5 is stabilized by TRMT112. Nucleic Acids Res.

[15] Pendleton K.E., Chen B., Liu K. (2017). The U6 snRNA m^6^A methyltransferase METTL16 regulates SAM synthetase intron retention. Cell.

[16] Wang X., Feng J., Xue Y. (2016). Structural basis of N^6^-adenosine methylation by the METTL3-METTL14 complex. Nature.

[17] Ping X.L., Sun B.F., Wang L. (2014). Mammalian WTAP is a regulatory subunit of the RNA N6-methyladenosine methyltransferase. Cell Res.

[18] Qian J.Y., Gao J., Sun X. (2019). KIAA1429 acts as an oncogenic factor in breast cancer by regulating CDK1 in an N6-methyladenosine-independent manner. Oncogene.

[19] Yue Y., Liu J., Cui X. (2018). VIRMA mediates preferential m^6^A mRNA methylation in 3'UTR and near stop codon and associates with alternative polyadenylation. Cell Discov.

[20] Coker H., Wei G., Moindrot B., Mohammed S., Nesterova T., Brockdorff N. (2020). The role of the Xist 5' m6A region and RBM15 in X chromosome inactivation. Wellcome Open Res.

[21] Patil D.P., Chen C.K., Pickering B.F. (2016). m^6^A RNA methylation promotes XIST-mediated transcriptional repression. Nature.

[22] Ma H., Wang X., Cai J. (2019). N6-Methyladenosine methyltransferase ZCCHC4 mediates ribosomal RNA methylation. Nat Chem Biol.

[23] Wen J., Lv R., Ma H. (2018). Zc3h13 regulates nuclear RNA m^6^A methylation and mouse embryonic stem cell self-renewal. Mol Cell.

[24] Zhang M., Zhang Y., Ma J. (2015). The demethylase activity of FTO (fat mass and obesity associated protein) is required for preadipocyte differentiation. PLoS One.

[25] Gerken T., Girard C.A., Tung Y.L. (2007). The obesity-associated FTO gene encodes a 2-oxoglutarate-dependent nucleic acid demethylase. Science.

[26] Aravind L., Koonin E.V. (2001). The DNA-repair protein AlkB, EGL-9, and leprecan define new families of 2-oxoglutarate- and iron-dependent dioxygenases. Genome Biol.

[27] Tsujikawa K., Koike K., Kitae K. (2007). Expression and sub-cellular localization of human ABH family molecules. J Cell Mol Med.

[28] Aik W., Scotti J.S., Choi H. (2014). Structure of human RNA N^6^-methyladenine demethylase ALKBH5 provides insights into its mechanisms of nucleic acid recognition and demethylation. Nucleic Acids Res.

[29] Ueda Y., Ooshio I., Fusamae Y. (2017). AlkB homolog 3-mediated tRNA demethylation promotes protein synthesis in cancer cells. Sci Rep.

[30] Xu C., Wang X., Liu K. (2014). Structural basis for selective binding of m6A RNA by the YTHDC1 YTH domain. Nat Chem Biol.

[31] Pan F., Hüttelmaier S., Singer R.H., Gu W. (2007). ZBP2 facilitates binding of ZBP1 to beta-actin mRNA during transcription. Mol Cell Biol.

[32] Degrauwe N., Suvà M.L., Janiszewska M., Riggi N., Stamenkovic I. (2016). IMPs: an RNA-binding protein family that provides a link between stem cell maintenance in normal development and cancer. Genes Dev.

[33] Geuens T., Bouhy D., Timmerman V. (2016). The hnRNP family: insights into their role in health and disease. Hum Genet.

[34] Meyer K.D., Patil D.P., Zhou J. (2015). 5' UTR m^6^A promotes cap-independent translation. Cell.

[35] Wu R., Li A., Sun B. (2019). A novel m^6^A reader Prrc2a controls oligodendroglial specification and myelination. Cell Res.

[36] Zhang F., Kang Y., Wang M. (2018). Fragile X mental retardation protein modulates the stability of its m6A-marked messenger RNA targets. Hum Mol Genet.

[37] Moor R.M., Dai Y., Lee C., Jr J.F. (1998). Oocyte maturation and embryonic failure. Hum Reprod Update.

[38] Fortune J.E., Cushman R.A., Wahl C.M., Kito S. (2000). The primordial to primary follicle transition. Mol Cell Endocrinol.

[39] Coticchio G., Dal Canto M., Mignini Renzini M. (2015). Oocyte maturation: gamete-somatic cells interactions, meiotic resumption, cytoskeletal dynamics and cytoplasmic reorganization. Hum Reprod Update.

[40] Yao H., Gao C.C., Zhang D. (2023). scm^6^A-seq reveals single-cell landscapes of the dynamic m^6^A during oocyte maturation and early embryonic development. Nat Commun.

[41] Mu H., Zhang T., Yang Y. (2021). METTL3-mediated mRNA N6-methyladenosine is required for oocyte and follicle development in mice. Cell Death Dis.

[42] Zhu Y., Wu W., Chen S. (2022). Mettl3 downregulation in germinal vesicle oocytes inhibits mRNA decay and the first polar body extrusion during maturation. Biol Reprod.

[43] Wu Y., Xu X., Qi M. (2022). N6-methyladenosine regulates maternal RNA maintenance in oocytes and timely RNA decay during mouse maternal-to-zygotic transition. Nat Cell Biol.

[44] Sui X., Hu Y., Ren C. (2020). METTL3-mediated m^6^A is required for murine oocyte maturation and maternal-to-zygotic transition. Cell Cycle.

[45] Yu X.X., Liu Y.H., Liu X.M. (2018). Ascorbic acid induces global epigenetic reprogramming to promote meiotic maturation and developmental competence of porcine oocytes. Sci Rep.

[46] Hu Y., Ouyang Z., Sui X. (2020). Oocyte competence is maintained by m^6^A methyltransferase KIAA1429-mediated RNA metabolism during mouse follicular development. Cell Death Differ.

[47] Wei J., Yu X., Yang L. (2022). FTO mediates LINE1 m^6^A demethylation and chromatin regulation in mESCs and mouse development. Science.

[48] Kasowitz S.D., Ma J., Anderson S.J. (2018). Nuclear m6A reader YTHDC1 regulates alternative polyadenylation and splicing during mouse oocyte development. PLoS Genet.

[49] Zeng M., Dai X., Liang Z. (2020). Critical roles of mRNA m^6^A modification and YTHDC2 expression for meiotic initiation and progression in female germ cells. Gene.

[50] Canipari R. (2000). Oocyte: granulosa cell interactions. Hum Reprod Update.

[51] Liu C., Li L., Yang B. (2022). Transcriptome-wide N6-methyladenine methylation in granulosa cells of women with decreased ovarian reserve. BMC Genom.

[52] Sun X., Zhang Y., Hu Y. (2021). Decreased expression of m^6^A demethylase FTO in ovarian aging. Arch Gynecol Obstet.

[53] Ding C., Zou Q., Ding J. (2018). Increased N6-methyladenosine causes infertility is associated with FTO expression. J Cell Physiol.

[54] Jiang Z.X., Wang Y.N., Li Z.Y. (2021). The m6A mRNA demethylase FTO in granulosa cells retards FOS-dependent ovarian aging. Cell Death Dis.

[55] Ding H., Li Z., Li X. (2022). FTO alleviates CdCl_2_-induced apoptosis and oxidative stress via the AKT/Nrf2 pathway in bovine granulosa cells. Int J Mol Sci.

[56] Mu H., Cai S., Wang X. (2022). RNA binding protein IGF_2_BP_1_ meditates oxidative stress-induced granulosa cell dysfunction by regulating MDM2 mRNA stability in an m^6^A-dependent manner. Redox Biol.

[57] Liu Z., Zhou L., Li D. (2023). N6-methyladenosine methyltransferase METTL3 modulates the cell cycle of granulosa cells via CCND1 and AURKB in Haimen goats. FASEB J.

[58] Li Z., Ruan Z., Feng Y. (2023). METTL3-mediated m6A methylation regulates granulosa cells autophagy during follicular atresia in pig ovaries. Theriogenology.

[59] Liu K., He X., Huang J. (2023). Short-chain fatty acid-butyric acid ameliorates granulosa cells inflammation through regulating METTL3-mediated N6-methyladenosine modification of FOSL2 in polycystic ovarian syndrome. Clin Epigenetics.

[60] Zhao S., Lu J., Chen Y., Wang Z., Cao J., Dong Y. (2021). Exploration of the potential roles of m6A regulators in the uterus in pregnancy and infertility. J Reprod Immunol.

[61] Sun Y., Zhang X.C., Li M.D. (2023). METTL3 promotes proliferation of goat endometrial epithelial cells by regulating CTGF in an m6A-dependent manner. Biol Reprod.

[62] Kobayashi R., Kawabata-Iwakawa R., Terakawa J. (2023). Aberrant activation of estrogen receptor-α signaling in Mettl14-deficient uteri impairs embryo implantation. FASEB J.

[63] Wan S., Sun Y., Zong J. (2023). METTL3-dependent m^6^A methylation facilitates uterine receptivity and female fertility via balancing estrogen and progesterone signaling. Cell Death Dis.

[64] Zheng Z.H., Zhang G.L., Jiang R.F. (2023). METTL3 is essential for normal progesterone signaling during embryo implantation via m^6^A-mediated translation control of progesterone receptor. Proc Natl Acad Sci USA.

[65] Xue P., Zhou W., Fan W. (2021). Increased METTL3-mediated m^6^A methylation inhibits embryo implantation by repressing HOXA10 expression in recurrent implantation failure. Reprod Biol Endocrinol.

[66] Abu-Raya B., Michalski C., Sadarangani M., Lavoie P.M. (2020). Maternal immunological adaptation during normal pregnancy. Front Immunol.

[67] Wang F., Jia W., Fan M. (2021). Single-cell immune landscape of human recurrent miscarriage. Genom Proteom Bioinform.

[68] Zhang X., Wei H. (2021). Role of decidual natural killer cells in human pregnancy and related pregnancy complications. Front Immunol.

[69] Fu B., Zhou Y., Ni X. (2017). Natural killer cells promote fetal development through the secretion of growth-promoting factors. Immunity.

[70] Ma S., Yan J., Barr T. (2021). The RNA m6A reader YTHDF2 controls NK cell antitumor and antiviral immunity. J Exp Med.

[71] Song H., Song J., Cheng M. (2021). METTL3-mediated m^6^A RNA methylation promotes the anti-tumour immunity of natural killer cells. Nat Commun.

[72] Kim S.M., Oh S.C., Lee S.Y., Kong L.Z., Lee J.H., Kim T.D. (2023). FTO negatively regulates the cytotoxic activity of natural killer cells. EMBO Rep.

[73] Tong J., Cao G., Zhang T. (2018). m^6^A mRNA methylation sustains Treg suppressive functions. Cell Res.

[74] Liu Y., Yuan Y., Zhou Z. (2022). Mettl14-mediated m6A modification enhances the function of Foxp3^+^ regulatory T cells and promotes allograft acceptance. Front Immunol.

[75] Chen J., Xu C., Yang K. (2023). Inhibition of ALKBH5 attenuates I/R-induced renal injury in male mice by promoting Ccl28 m6A modification and increasing Treg recruitment. Nat Commun.

[76] Zhang L., Dou X., Zheng Z. (2023). YTHDF2/m^6^ A/NF-κB axis controls anti-tumor immunity by regulating intratumoral Tregs. EMBO J.

[77] Boutari C., Pappas P.D., Mintziori G. (2020). The effect of underweight on female and male reproduction. Metabolism.

[78] Silvestris E., de Pergola G., Rosania R., Loverro G. (2018). Obesity as disruptor of the female fertility. Reprod Biol Endocrinol.

[79] Fulton S., Décarie-Spain L., Fioramonti X., Guiard B., Nakajima S. (2022). The menace of obesity to depression and anxiety prevalence. Trends Endocrinol Metabol.

[80] Wang Y., Wang Y., Gu J., Su T., Gu X., Feng Y. (2022). The role of RNA m6A methylation in lipid metabolism. Front Endocrinol.

[81] Claussnitzer M., Dankel S.N., Kim K.H. (2015). FTO obesity variant circuitry and adipocyte browning in humans. N Engl J Med.

[82] Zhao X., Yang Y., Sun B.F. (2014). FTO-dependent demethylation of N6-methyladenosine regulates mRNA splicing and is required for adipogenesis. Cell Res.

[83] Wang X., Wu R., Liu Y. (2020). m^6^A mRNA methylation controls autophagy and adipogenesis by targeting *Atg5* and *Atg7*. Autophagy.

[84] Li Y., Zhang Y., Zhang T. (2023). RNA M^6^ a methylation regulates glycolysis of beige fat and contributes to systemic metabolic homeostasis. Adv Sci (Weinh).

[85] Wang Y., Gao M., Zhu F. (2020). METTL3 is essential for postnatal development of brown adipose tissue and energy expenditure in mice. Nat Commun.

[86] Hubacek J.A., Dlouha L., Adamkova V. (2023). Genetic risk score is associated with T2DM and diabetes complications risks. Gene.

[87] Li X., Jiang Y., Sun X., Wu Y., Chen Z. (2021). METTL3 is required for maintaining β-cell function. Metabolism.

[88] Li X., Yang Y., Chen Z. (2023). Downregulation of the m^6^A reader protein YTHDC1 leads to islet β-cell failure and diabetes. Metabolism.

[89] Liu S., Xiu J., Zhu C. (2021). Fat mass and obesity-associated protein regulates RNA methylation associated with depression-like behavior in mice. Nat Commun.

[90] Khizroeva J., Nalli C., Bitsadze V. (2019). Infertility in women with systemic autoimmune diseases. Best Pract Res Clin Endocrinol Metabol.

[91] Paul van Trotsenburg A.S. (2020). Management of neonates born to mothers with thyroid dysfunction, and points for attention during pregnancy. Best Pract Res Clin Endocrinol Metabol.

[92] Mo K., Chu Y., Liu Y. (2023). Targeting hnRNPC suppresses thyroid follicular epithelial cell apoptosis and necroptosis through m^6^A-modified ATF4 in autoimmune thyroid disease. Pharmacol Res.

[93] Ni Z., Sun S., Bi Y. (2020). Correlation of fecal metabolomics and gut microbiota in mice with endometriosis. Am J Reprod Immunol.

[94] Gruber T.M., Mechsner S. (2021). Pathogenesis of endometriosis: the origin of pain and subfertility. Cells.

[95] Li X., Xiong W., Long X. (2021). Inhibition of METTL3/m6A/miR126 promotes the migration and invasion of endometrial stromal cells in endometriosis. Biol Reprod.

[96] Zhang Q.C. (2023). METTL3 is aberrantly expressed in endometriosis and suppresses proliferation, invasion, and migration of endometrial stromal cells. Kaohsiung J Med Sci.

[97] Gou Y., Wang H., Wang T. (2023). Ectopic endometriotic stromal cells-derived lactate induces M2 macrophage polarization via Mettl3/Trib1/ERK/STAT3 signalling pathway in endometriosis. Immunology.

[98] Wang X., Wang J., Zhao X. (2023). METTL3-mediated m6A modification of SIRT1 mRNA inhibits progression of endometriosis by cellular senescence enhancing. J Transl Med.

[99] Shen L., Zhang C., Zhang Y., Yang Y. (2023). METTL3 and METTL14-mediated N6-methyladenosine modification promotes cell proliferation and invasion in a model of endometriosis. Reprod Biomed Online.

[100] Zhao S., Zhang B., Yuan H. (2022). IGF_2_BP_2_ promotes the progression of ovarian endometriosis by regulating m6A-modified MEIS2 and GATA6. Int J Biochem Cell Biol.

[101] Wang H., Liang Z., Gou Y. (2022). FTO-dependent N6-Methyladenosine regulates the progression of endometriosis via the ATG5/PKM2 Axis. Cell Signal.

[102] Li X., Jin J., Long X. (2023). METTL3-regulated m6A modification impairs the decidualization of endometrial stromal cells by regulating YTHDF2-mediated degradation of FOXO1 mRNA in endometriosis-related infertility. Reprod Biol Endocrinol.

[103] Joham A.E., Norman R.J., Stener-Victorin E. (2022). Polycystic ovary syndrome. Lancet Diabetes Endocrinol.

[104] Li Y., Chen C., Ma Y. (2019). Multi-system reproductive metabolic disorder: significance for the pathogenesis and therapy of polycystic ovary syndrome (PCOS). Life Sci.

[105] Sun X., Lu J., Li H., Huang B. (2022). The role of m^6^A on female reproduction and fertility: from gonad development to ovarian aging. Front Cell Dev Biol.

[106] Zhang S., Deng W., Liu Q., Wang P., Yang W., Ni W. (2020). Altered m^6^ A modification is involved in up-regulated expression of FOXO3 in luteinized granulosa cells of Non-obese polycystic ovary syndrome patients. J Cell Mol Med.

[107] Haouzi D., Assou S., Monzo C., Vincens C., Dechaud H., Hamamah S. (2012). Altered gene expression profile in cumulus cells of mature MII oocytes from patients with polycystic ovary syndrome. Hum Reprod.

[108] Mikaeili S., Rashidi B.H., Safa M. (2016). Altered FoxO3 expression and apoptosis in granulosa cells of women with polycystic ovary syndrome. Arch Gynecol Obstet.

[109] Zhou L., Han X., Li W. (2022). N6-methyladenosine demethylase FTO induces the dysfunctions of ovarian granulosa cells by upregulating flotillin 2. Reprod Sci.

[110] Jing Y.X., Li H.X., Yue F. (2023). N6-methyladenosine demethylase FTO related to hyperandrogenism in PCOS via AKT pathway. Gynecol Endocrinol.

[111] Rull K., Nagirnaja L., Laan M. (2012). Genetics of recurrent miscarriage: challenges, current knowledge, future directions. Front Genet.

[112] Mi C., Chen W., Liang T. (2022). lnc-HZ05 regulates BPDE-inhibited human trophoblast cell proliferation and affects the occurrence of miscarriage by directly binding with miR-hz05. Cell Biol Toxicol.

[113] Ye Y., Jiang S., Du T. (2023). Correction: environmental pollutant benzo[a]pyrene upregulated long non-coding RNA HZ07 inhibits trophoblast cell migration by inactivating PI3K/AKT/MMP2 signaling pathway in recurrent pregnancy loss. Reprod Sci.

[114] Li X.C., Jin F., Wang B.Y., Yin X.J., Hong W., Tian F.J. (2019). The m6A demethylase ALKBH5 controls trophoblast invasion at the maternal-fetal interface by regulating the stability of *CYR61* mRNA. Theranostics.

[115] Huang N., Gao Y., Zhang M. (2022). METTL3-mediated m^6^A RNA methylation of ZBTB4 interferes with trophoblast invasion and maybe involved in RSA. Front Cell Dev Biol.

[116] Zhao Y., Sun J., Jin L. (2022). The N6-methyladenosine regulator *ALKBH5* mediated stromal cell-macrophage interaction via VEGF signaling to promote recurrent spontaneous abortion: a bioinformatic and *in vitro* study. Int J Mol Sci.

[117] Zheng Q., Yang F., Gan H., Jin L. (2022). Hypoxia induced ALKBH5 prevents spontaneous abortion by mediating m^6^A-demethylation of SMAD1/5 mRNAs. Biochim Biophys Acta Mol Cell Res.

[118] Xu Z., Tian P., Guo J. (2021). lnc-HZ01 with m6A RNA methylation inhibits human trophoblast cell proliferation and induces miscarriage by up-regulating BPDE-activated lnc-HZ01/MXD1 positive feedback loop. Sci Total Environ.

[119] Dai M., Huang W., Huang X. (2023). BPDE, the migration and invasion of human trophoblast cells, and occurrence of miscarriage in humans: roles of a novel *lncRNA-HZ09*. Environ Health Perspect.

[120] Wang R., Xu X., Yang J. (2023). BPDE exposure promotes trophoblast cell pyroptosis and induces miscarriage by up-regulating lnc-HZ14/ZBP1/NLRP3 axis. J Hazard Mater.

[121] Qiu L., Jing Q., Li Y., Han J. (2023). RNA modification: mechanisms and therapeutic targets. Mol Biomed.

